# Rscreenorm: normalization of CRISPR and siRNA screen data for more reproducible hit selection

**DOI:** 10.1186/s12859-018-2306-z

**Published:** 2018-08-20

**Authors:** Costa Bachas, Jasmina Hodzic, Johannes C. van der Mijn, Chantal Stoepker, Henk M. W. Verheul, Rob M. F. Wolthuis, Emanuela Felley-Bosco, Wessel N. van Wieringen, Victor W. van Beusechem, Ruud H. Brakenhoff, Renée X. de Menezes

**Affiliations:** 1 Department of Otolaryngology - Head and Neck Surgery, Amsterdam UMC, Vrije Universiteit Amsterdam, De Boelelaan 1117, Amsterdam, 1081 HV The Netherlands; 2 Department of Epidemiology and Biostatistics, Amsterdam UMC, Vrije Universiteit Amsterdam, Amsterdam, 1007 MB The Netherlands; 3 Department of Medical Oncology, Amsterdam UMC, Vrije Universiteit Amsterdam, De Boelelaan 1117, Amsterdam, 1081 HV The Netherlands; 4 Division of Tumor Biology and Immunology, Netherlands Cancer Institute, Amsterdam, The Netherlands; 5 Section of Oncogenetics, Department of Clinical Genetics, Amsterdam UMC, Vrije Universiteit Amsterdam, De Boelelaan 1118, Amsterdam, 1081 HV The Netherlands; 6 Laboratory of Molecular Oncology, University Hospital Zürich, Zürich, Switzerland; 7 Department of Mathematics, VU University, Amsterdam, The Netherlands

**Keywords:** Functional genomics, Reproducibility, Normalization

## Abstract

**Background:**

Reproducibility of hits from independent CRISPR or siRNA screens is poor. This is partly due to data normalization primarily addressing technical variability within independent screens, and not the technical differences between them.

**Results:**

We present “rscreenorm”, a method that standardizes the functional data ranges between screens using assay controls, and subsequently performs a piecewise-linear normalization to make data distributions across all screens comparable. In simulation studies, rscreenorm reduces false positives. Using two multiple-cell lines siRNA screens, rscreenorm increased reproducibility between 27 and 62% for hits, and up to 5-fold for non-hits. Using publicly available CRISPR-Cas screen data, application of commonly used median centering yields merely 34% of overlapping hits, in contrast with rscreenorm yielding 84% of overlapping hits. Furthermore, rscreenorm yielded at most 8% discordant results, whilst median-centering yielded as much as 55%.

**Conclusions:**

Rscreenorm yields more consistent results and keeps false positive rates under control, improving reproducibility of genetic screens data analysis from multiple cell lines.

**Electronic supplementary material:**

The online version of this article (10.1186/s12859-018-2306-z) contains supplementary material, which is available to authorized users.

## Background

Genetic screens for genome-wide perturbation of genes are widely used in cell biology studies and drug target discovery [1]. Unfortunately, independent study results show limited reproducibility, often thought to arise from extensive off-target effects and variable knock-down or knock-out efficiencies [2]. However, studies also differ intrinsically with respect to experimental design, readouts, assay lengths, transfection efficiencies, and data processing. For example, effects of individual gene perturbations, represented by different small-interference RNAs (siRNAs) or guide RNAs (gRNAs), may be studied by assessing cell depletion from a mixture of library features (pooled screening format) or in separate wells of a micro-titer plate (arrayed screening format). All these aspects can introduce technical noise in the data, which must be corrected in order to yield reliable results. Here we focus on screens using RNA interference (RNAi) [3] or genome editing techniques [4] such as the CRISPR-Cas system [5–7].

Correcting technical noise is particularly difficult in studies that involve both technical replicates as well as biological ones, for example involving multiple cell lines and/or treatment effects. Statistical methods that correct for undesired variation *within* individual screens are available [8, 9]. However, technical variability *between* datasets remains, yielding for example cell line-dependent functional ranges. Consequently, similar data values may represent different phenotypes in different data sets, hampering reproducibility of genetic screening results [10–12]. Most currently available methods merely center each replicate regardless of assay controls, which fails to guarantee that centered values represent similar phenotypes. In particular, differences between biological replicates, that may be observed as shifts between the data distributions of two cell lines of the same tumor type, are removed by replicate centering.

We propose a non-parametric normalization called “rscreenorm”, a smart analysis pipeline that prepares data of multiple and independently collected genetic screens for statistical analysis by making their functional ranges and distributions comparable. Rscreenorm reduces false positive rates in hit lists, as we show in a simulation study, in our siRNA screen data example involving genome-wide as well as validation screens, and in publicly available CRISPR-Cas screen data.

## Methods

### Motivation

Genetic screens yield functional data, in that they measure a phenotype yielded by gene perturbations introduced by RNA interference (RNAi) or genome editing. As such, an essential part of these data are negative and positive assay controls, which yield reference measurements for normal and altered phenotypes, respectively.

A typical study design may involve both biological as well as technical replicates. We call biological replicates those corresponding to different cell lines of the same tumor type. In contrast, technical replicates typically correspond to the same individual cell lines and condition. In what follows, “replicates” will refer to technical replicates, whilst “screens” will refer to biological replicates involving different cell lines.

**Fig. 1:**
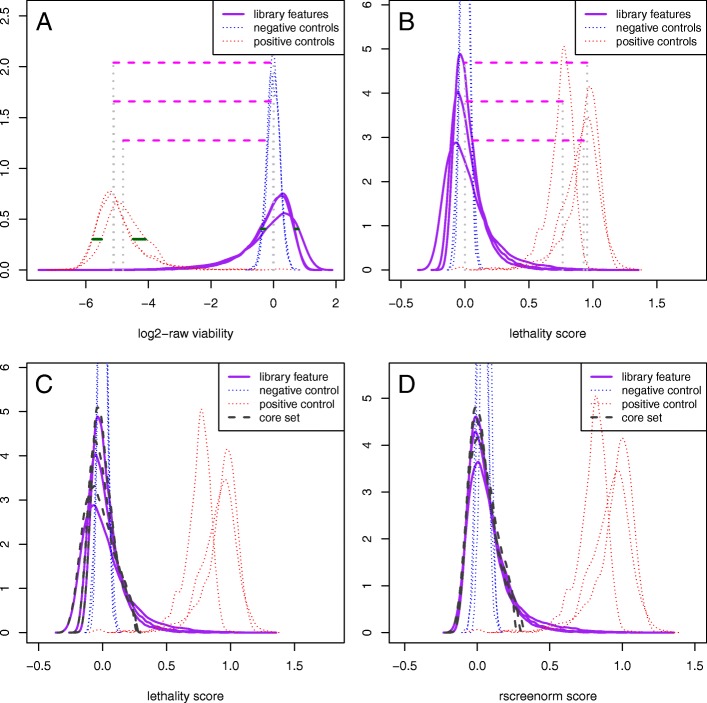
Overview of methods steps. Schematic overview of rscreenorm steps. Illustrations use the arrayed whole-genome lethality siRNA screen on cell line 786-O (3 replicates). **a** Raw (log2-transformed) viability empirical distributions, separately for library features, negative and positive controls, with between-replicates differences in functional range and data distributions illustrated by pink-dashed and green-solid lines, respectively. **b** Densities of lethality scores, representing the phenotype relative to the assay controls. These make data values and functional ranges more comparable, but differences between data distributions (in purple lines) remain. **c** a core set of lethality scores (dark-gray dashed lines) is chosen per replicate. **d** Distributions of rscreenorm scores, where most differences between replicates have been corrected for

Assay controls typically display variation across replicates and, in case of arrayed screens, across the multiple plates of screen replicates. This means that reference values for normal and lethal phenotypes, which define the functional range of measurements, may be influenced by experimental design and, specifically, may vary across replicates, as illustrated in Fig. 1a. We will take into account assay controls’ viabilities during preprocessing to make data from multiple screens comparable, facilitating interpretation and analysis.

The method involves the following steps:

- compute lethality scores;
- compute core sets of values per replicate;
- compute and normalize quantiles for the core sets;
- extrapolate normalization to the core sets;
- extend normalization to scores outside the core set.

Step i) makes functional ranges comparable across replicates, and steps ii)-v) normalize viability values by using a comparable part of the measurements’ distributions, while allowing for some screens to have higher proportions of extreme data values. An overview of the method is given in Fig. 1 and, in the following subsections, we will explain each step in detail.

In what follows, we focus on cell viability as the phenotype of interest and we will refer to it as “phenotype” and “cell viability” interchangeably, but we point out that our method can be used for any given read-out of interest. For any replicate *k* we will represent by *Z*_*ik*_ the cell viability measured for an interference (siRNA) or a genome modification *i* as part of a larger library of perturbations (*i*=1,…,*n*). In such cases, per-replicate cell viability values {*Z*_*ik*_}_*i*_ must be interpreted in the context of cell viability measured for replicate-specific assay controls, negative $\left \{ Z^{N}_{k} \right \}$ representing normal viability, and positive $\left \{ Z^{P}_{k} \right \}$ representing loss of viability, the latter indicating a lethal phenotype.

### i) Compute lethality scores

Per replicate of a screen, we compute a *lethality score* for each data point, expressing differences between library features and the median of negative controls per replicate, relative to the difference between the medians of negative and positive controls. In general, for a given library feature *i* and replicate *k*, this can be written as:

1 $$ {} Z_{ik} \,=\, \frac{\text{observation for feature} i,k \,-\, \text{median negative controls}} {\text{median positive controls} \,-\, \text{median negative controls}}  $$

**Fig. 2:**
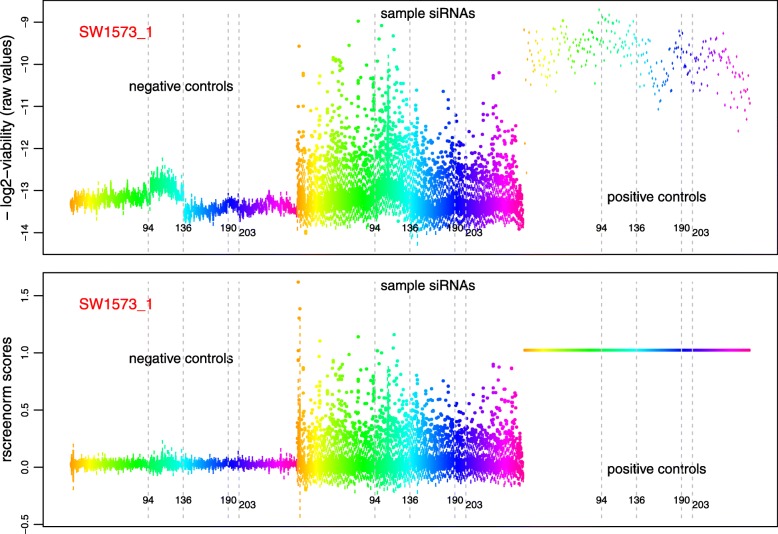
Plate effects before and after rscreenorm. Removal of plate effects per replicate of an arrayed screen by rscreenorm. Boxplots of log2-raw viability values (top) and rscreenorm scores (bottom) for replicate 1 of cell line SW1573. Plates 1-272 (from left to right) are displayed with the same colours for negative controls (left boxes), sample siRNAs (middle boxes) and positive controls (right boxes). Plate 1 is coloured orange (left-most box for each well type), and plate 272 is coloured pink (right-most box). The top graph displays -log2-raw data, to make boxes in both plots easily comparable – so in all cases, values indicating more lethality are displayed higher than those corresponding to more viability. Vertical axis: top graph has -log2-viability; bottom graph has rscreenorm scores (computed using the log2-intensity read-outs)

Resulting scores represent the phenotype (cell viability) on the same scale, for all replicates of all screens. Features with phenotypes similar to that of negative controls yield scores around zero, whilst those with phenotype as lethal as positive controls get scores around 1 (Fig. 1a-b). Thus, lethality scores are standardized measurements that represent the phenotype on similar functional range across replicates and cell lines (Additional file 1: Figure S1 and S2). In case of arrayed screens, this range may be computed per plate, thus using medians of controls per plate, naturally correcting for plate effects (Fig. 2).

While lethality scores make functional ranges comparable, considerable variability between screen replicates still remains (Fig. 1b, purple lines). To reduce this, we first choose per replicate a core of lethality scores representing a range of phenotypes observed for all screens in similar proportions, as detailed in the next subsection.

### ii) Compute core sets of values per replicate

Lethality scores represent standardized cell viabilities, produced by a mixture of biological signal (true viability measurement) and technical noise. It is reasonable to expect that each screen yields a range of lethality scores that represent largely overlapping phenotypes with other screens, except for extreme scores. Screens with more a higher proportion of extreme lethality scores, display data distributions with heavier upper tails. As such, one would expect that lethality scores distributions for different screens would be similar in their core, except possibly for their tails, after technical noise is corrected away. Thus, per replicate we will define the core set of lethality scores as the values representing the range of phenotypes likely to be observed in similar proportions across screens, and thus excluding the upper tails. Note that some genetic screens can yield extreme phenotypes corresponding to both more lethality as well as increased proliferation, in which case a core set excluding both tails is preferable – we indicate how such a core set can be build in the Additional file 1.

Per replicate, the distribution core is defined as the set of scores representing the part of their distribution likely to be similar, excluding the upper tail (see Fig. 1c and Additional file 1). This makes sure that normalization does not correct away possibly different proportions of hits on the upper tail.

Specifically, per replicate *k* the core set $\left \{Z_{ik}^{\mathrm {c}}\right \}$ of lethality scores is formed by the scores satisfying *Z*_*ik*_≤*Zkα*_*k*_, where $Z^{\alpha _{k}}_{k}$ represents the percentile of the lethality scores that is smaller than *α*_*k*_% of the scores of replicate *k*, for some *α*_*k*_ such that 0<*α*≤1. In particular, if *α*_*k*_=1, all lethality scores are included in the core set. Note that the core set is a set of *values*, not of features.

In order to construct such a core set, *α*_*k*_ needs to be fixed for each replicate. In a context where whole-genome screens are produced for cell lines under the same condition, it is reasonable to expect that between 90 and 95% of the entire phenotype range will be represented in all screens in roughly the same proportions, so that a good option is to choose *α*_*k*_=0.95 for all replicates *k*=1,…,*K*. However, if cell lines are screened under different conditions, say before and after treatment, then *α*_*k*_ may be chosen according to the expected treatment effect. A treatment effect expected to yield increased susceptibility to genetic interference, for example leading to 20% lethal hits, would require *α*_*k*_=0.95 for the untreated cell lines, and *α*_*k*_=0.80 for the treated ones.

In some cases, there is no natural way of choosing *α*_*k*_. Then we suggest constructing the core set using the distances between lethality scores and the assay controls. This takes the phenotype into account, yielding interpretable core sets. See the Additional file 1 for details.

Note that core sets may vary in size, in particular when different proportions of scores on the tails are expected. Therefore, the method must be able to consider different set sizes.

After choosing core sets, we normalize the distributions of lethality scores across screens and replicates to make them comparable.

### iii) Compute and normalize quantiles for the core sets

We apply quantile normalization to the core set distributions only, so as to preserve differences in the tails of each replicate. However, this requires same size sets, whilst core sets may vary in size. To overcome this, we represent the range of values in the core set by a fixed set of quantiles. Specifically, this involves replacing the core lethality scores of each replicate by their {*j*/1000, *j*=1,…,1000} quantiles, represented by $\left \{Z_{jk}^{q}\right \}_{j=1}^{1000}$, where $Z_{j-1,k}^{q} < Z_{jk}^{q}$ and *k*,*j* represent the replicate and the quantile, respectively.

We subsequently apply quantile normalization by replacing each core set quantile by the average of core set quantiles across all samples:

2 $$ \tilde{Z_{j}^{q}} = \frac{1}{K} \sum_{k=1}^{K} Z_{jk}^{q}, \quad j=1,\ldots,1000,  $$

for each given *j*, where *K* represents the total number of replicates from all data sets together. After normalizing the $\left \{Z_{jk}^{q}\right \}_{j=1}^{1000}$, the normalization needs to be extended to all values in the core sets.

### iv) Extrapolate normalization to the core sets

A linear regression between original and normalized quantiles is used to compute normalizing factors for the scores in the core set. Specifically, we first compute:

3 $$ \tilde{Z_{jk}^{q}} = \alpha_{k} + \beta_{k} Z_{jk}^{q} + \epsilon_{i},\quad j=1,\ldots,1000,  $$

for each *k*=1,…,*K*, yielding $\hat {\alpha _{k}}, \hat {\beta _{k}}$, for each replicate *k*. This essentially re-expresses the data rescaling that resulted from the quantile normalization as a linear regression, enabling us to apply it to the entire core set of values.

Subsequently, for each score *Z*_*ik*_ within the core set $\left \{Z_{ik}^{\mathrm {c}}\right \}$, the normalized value $Z_{ik}^{\text {norm}}$ can be computed using:

4 $$ Z_{ik}^{\text{norm}} = \hat{\alpha_{k}}+ \hat{\beta_{k}} Z_{ik}, \quad k=1,\ldots,K.  $$

### v) Extrapolate normalization to scores outside the core sets

Scores outside the core set are shifted by the same amount as the nearest score in the core. Let us represent by $Z_{k}^{\text {min}}, Z_{k}^{\text {max}}$ the minimum and maximum scores in the core set $\left \{Z_{ik}^{\mathrm {c}}\right \}$, for each given replicate *k*. Then, for any $Z_{ik}>Z_{k}^{\text {max}}$, the normalized score becomes:

5 $$ Z_{ik}^{\text{norm}} = \hat{\alpha_{k}}+ \left(\hat{\beta_{k}}-1\right) Z_{k}^{\text{max}} + Z_{ik}, \quad k=1,\ldots,K.  $$

Similarly, for scores satisfying $Z_{ik}<Z_{k}^{\text {min}}$, the normalized score becomes:

6 $$ Z_{ik}^{\text{norm}} = \hat{\alpha_{k}}+ \left(\hat{\beta_{k}}+1\right) Z_{k}^{\text{min}} - Z_{ik}, \quad k=1,\ldots,K.  $$

This normalization results in a piecewise-linear transformation of the lethality scores, since it consists of applying separate linear transformations to values within and outside the core set range. With noise as much as possible eliminated and phenotypic effects preserved, rscreenorm yields scores that are comparable in terms of the core of their empirical distributions across replicates (Fig. 1d).

## Results

### Simulation study

We ran a simulation study involving 6 cell lines, each being screened in triplicate. Independently per replicate, we generate 1000 lethality scores for library features, as well as 200 for each of negative and positive controls. Interest lies in finding library features that yield different phenotypes between cell lines. All entries in the data matrix are drawn from independent normal distributions: negative and positive controls with means 0 and 1, and both with standard deviation 0.1. Library features are drawn from a normal distribution with standard deviation 0.2, each with a different mean *μ*_*i*_(*i*=1,…,1000) such that $\mu _{i}\sim {\mathcal {B}}(2,6)$, per replicate. This beta distribution is asymmetric to the left, with expected value 2/8=0.25, so that most library features yield little lethality. The above describes the setup used where library features display no differential phenotype between cell lines in general, and will be referred as the “no effect” setup.

We also simulated data with 20% of library features displaying differential phenotype between cell lines. Specifically, in this setup 200 features had observations for cell lines 4, 5 and 6 generated with a mean of 0.5, which we will call the “group” effect. This will be referred as the “with effect” setup.

To make the data more realistic, in both setups we also introduced a stretch/contraction effect, which consisted in multiplying the means of all lethality scores (library features as well as positive controls) by a fixed value, per replicate. Constants used here are: 0.7 (cell lines 1, 4) and 1.4 (cell lines 3, 6). Data for cell lines 2, 5 was neither stretched nor contracted after simulation. The result is visible on the lethality scores distributions (Additional file 1: Figure S1: top-left and bottom-left graphs for no-effect and with-effect setups, respectively). Note that this effect is orthogonal to the group effect.

Rscreenorm corrects the stretch/contraction effect both in the no-effect setup (Additional file 1: Figure S1, top-middle graph), as well as in the with-effect setup (Additional file 1: Figure S1, bottom-middle graph). In the latter setup, the group effect is preserved, since the warm-coloured empirical data distributions (representing replicates of cell lines 4, 5 and 6) display a clear enrichment of lethal phenotypes in their upper tail, compared with the blue-green empirical data distributions(representing replicates of cell lines 1, 2 and 3).

Subsequently, we simulated 1000 datasets for each of the two setups above. Per dataset, a regression model was used to find features with a group effect, i.e. that displayed differential phenotype between cell lines {1,2,3} and {4,5,6}. This involved applying a Student’s t-test to the regression coefficient representing the group per library feature, and subsequently correcting the resulting *p*-values for multiple testing using the false discovery rate (FDR, [13]).

Rscreenorm yields results at or under the FDR-control level, whilst the lethality scores yield false positives well above that level. Indeed, in the no-effect setup, any feature found by the model is a false positive and, as such, their proportion should be around the FDR control level. This is indeed the case with all rscreenorm data results (green boxplots in top-right graph of Additional file 1: Figure S1). In the with-effect setup, rscreenorm yields conservative results, with false positive proportions below the FDR-control levels (green boxplots in bottom-right graph of Additional file 1: Figure S1). In both setups, results using non-normalized lethality scores yield many more false positives than the FDR-control level (blue boxplots in top- and bottom-right graphs of Additional file 1: Figure S1).

Had we used classic quantile normalization instead of rscreenorm, the group effect would have been corrected away, and we would have reduced the power to find true positives (Additional file 1: Figure S2).

The above simulation setup assumes that negative and positive controls behave as expected, yielding responses in accordance to their phenotypes. However, it is also of interest to understand how the method works when controls yield biased responses, which sometimes happens due to technical reasons. We evaluated this by including a bias on the positive controls’ means, which is orthogonal to the group effect as is the stretch effect (Additional file 1: Figure S3). Results showed that, when the bias leads to a larger overlap between positive controls and library features for at least some cell lines, using rscreenorm yields more true positives than when controls are ignored (Additional file 1: Figure S4). On the other hand, when the bias leads to a smaller overlap between positive controls and library features, ignoring the controls yields more true discoveries than when using rscreenorm, similarly to the case without bias. Note, however, that in all cases only rscreenorm yields the false discovery proportions within the expected range, whilst non-normalized lethality scores yield many more false discoveries than expected (Additional file 1: Figure S4).

More details about the setup and the analysis results can be found in Section 1 of the Additional file 1.

#### Example: siRNA screen data

**Fig. 3:**
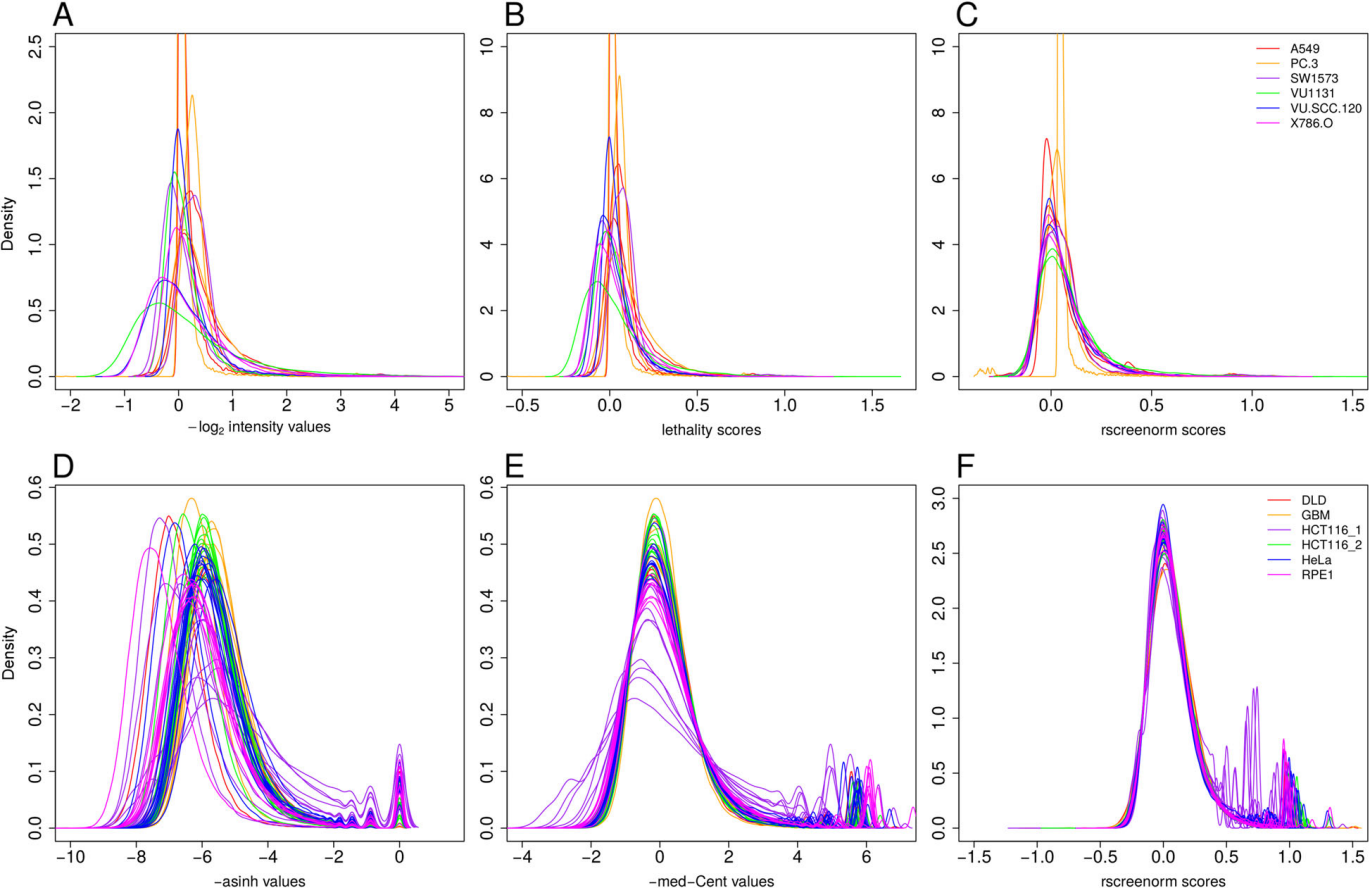
Data distributions before and after rscreenorm. Lethality scores’ and rscreenorm scores’ empirical distributions of data corresponding to multiple independent genetic screens. Upper panels show data of genome-wide siRNA screens, lower panels data of CRISPR-Cas screens data by Hart et al. [17]. Lines in all panels represent density values for library features, per replicate. **a** Log2-transformed viabilities corrected for plate effects, multiplied by -1 to facilitate visual comparison with lethality and rscreenorm scores. **b** Lethality scores prior to rscreenorm. **c** Rscreenorm scores. **d** Hyperbolic-arc sine transformed cell counts per guide RNA, multiplied by -1 to facilitate visual comparison with lethality and rscreenorm scores. **e** Lethality scores prior to rscreenorm. **f** Rscreenorm scores

We applied rscreenorm to genome-wide siRNA screens [14–16] data of 7 human cell lines (see Additional file 1). After centering each replicate around the negative controls’ median, we could see that (log2-) measured viabilities displayed considerable variability between cell lines: the viability range width represented by the difference between negative and positive controls’ medians varies between 0.7 for one replicate of cell line VU-SCC-120 and 5.1 for replicate 3 of cell line 786-O (Additional file 1: Figure S5). In addition, library siRNAs siUBB, siUBC and siPLK1 consistently displayed lethal phenotype across all cell lines and replicates, but yielded different log2-viabilities depending on the cell line: for example, for cell lines 786-O and VU1131 these values were below -4 for all replicates, whilst for SW1573 and VU-SCC-120 they were between -4 and -2 (Additional file 1: Figure S5). Furthermore, arrayed siRNA screens typically display a plate effect (top graph in Fig. 2). Here we point out that, if controls were ignored, the data would display much variability between 3 replicates and cell lines (Fig. 3a).

Remarkably, the plate effect influences measurements of a plate’s wells in a similar way, so that library siRNAs, negative and positive controls all display roughly the same effect per plate (top graph in Fig. 2). Since negative and positive controls yield the same phenotype on all plates, these can be used to make functional ranges comparable across plates. Indeed, if we consider plates 94–136, we notice that viabilities of all well types are shifted upwards by roughly similar amounts, and that this shift is corrected by rscreenorm (bottom graph in Fig. 2). A similar association between trends in viabilities of different well types is seen for plates 190–203.

Rscreenorm’s lethality scores already represent similar phenotypes with comparable values, but differences between replicates remain. Indeed, plate-specific functional ranges for lethality scores are comparable across plates and, thus, also between screens (Fig. 3b and Additional file 1: Figure S6). In particular, lethality scores for library siRNAs siUBB, siUBC and siPLK1 became much more comparable, being all between 0.5 and 1.5 (Additional file 1: Figure S6). While this makes functional ranges of different cell lines comparable, differences between empirical distributions of lethality scores for library siRNAs remain (Fig. 3b). We want to correct for those, while allowing for some cell lines to display a larger proportion of lethal hits, by means of heavier tails for the library siRNAs empirical distributions.

We first chose the core set scores for normalization by taking those lethality scores that were closer to negative than to positive controls, using *γ*=1 (see subsection 1.1 of the Additional file 1). This yielded varying inclusion proportions of lethality scores (Additional file 1: Figure S7) and, in some cases, the proportion achieved seemed too low. For this reason, instead we constructed core sets by taking 95% of the smallest lethality scores, which resulted in considerably less variation between replicates (Additional file 1: Figure S8), while preserving differences in lethal siRNA values across cell lines (Fig. 3a-c). In particular, plate-specific effects are corrected for, and functional ranges comparable (bottom graph in Fig. 2). In contrast, the often used robust z-scores, which center and standardize values per screen yielding per replicate mean and variance 0 and 1 respectively, leave plate effects unchanged (Additional file 1: Figure S9).

**Fig. 4:**
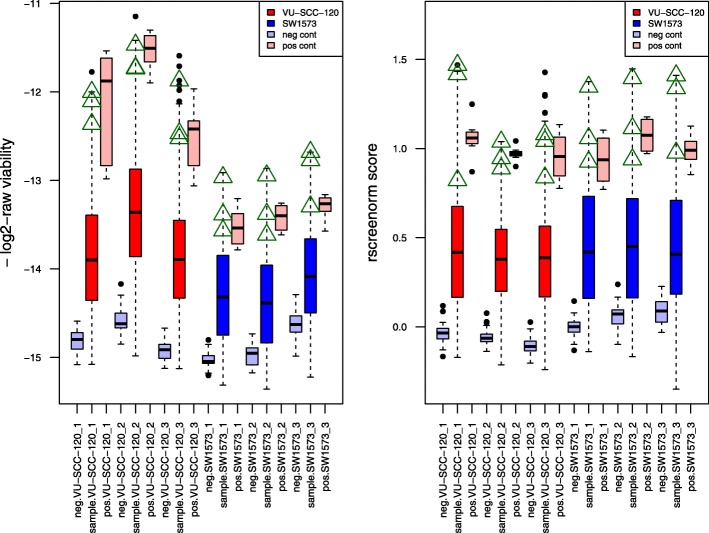
Validation siRNA screen data before and after rscreenorm. Boxplots for -log2-raw (left, where -log2 is used for better visual comparison with rscreenorm scores) and rscreenorm (right) data per replicate of cell lines VU-SCC-120 (library siRNAs in red) and SW1573 (library siRNAs in blue), and per well type (negative controls in light blue, positive controls in pink). Values for lethal library siRNAs siUBB, siUBC and siPLK1 are displayed as green triangles

Subsequent validation screens showed that rscreenorm yields better reproducibility of results than if robust z-scores had been used. Specifically, two of these cell lines (VU-SCC-120 and SW1573) were used in a secondary validation screen, based on 305 siRNAs then selected using their primary screen z-scores. Normalization using rscreenorm made again functional ranges more comparable, and yielded more similar values for lethal phenotypes (Fig. 4), which helps with later analyses that include both cell lines data in a single model. In particular, log2-raw data for cell line SW1573 shows compression compared with values for VU-SCC-120, and this is corrected by rscreenorm, also making values for lethal siRNAs comparable. Indeed, after using a regression model to compare the two cell lines based upon either robust z-scores or rscreenorm data, rscreenorm yielded a hit confirmation of 47% (91/194) of the primary screen hits, 27% more than with commonly used robust z-scores which only confirmed 37% (99/267) (Table 1). The agreement between results of the genome-wide (primary) and validation (secondary) screens, represented by the proportion of siRNAs with the same conclusion in both screens, also improved with rscreenorm: using robust z-scores only 34% (104/305) of the results were in agreement, whilst using rscreenorm this was 55% (169/305), representing a 62% increase (Table 1). For not-significant results, only 14% (5/38) of the robust z-scores’ primary screen not-significant siRNAs were also found to be not significant in the secondary screen, whilst this proportion is 5 times larger (70%, or 78/111) with rscreenorm.

**Table 1:** Numbers of significant and not-significant siRNAs from the primary whole genome siRNA screen (columns) and a secondary validation screen (rows) on two cell lines using an empirical Bayes model (FDR ≤ 0.05) on either robust z-scores or rscreenorm data

		Primary screen		
Robust z-scores		Not significant	Significant	Total
Secondary screen	Not significant	5	168	173
	Significant	33	99	132
Total		38	267	305
Rscreenorm				
Secondary screen	Not significant	78	103	181
	Significant	33	91	124
Total		111	194	305

The screens involved 21121 and 305 siRNAs each, respectively

We conclude that rscreenorm yields better agreement between hits/non-hits lists, obtained with our whole-genome and validation siRNA screens.

#### Example: CRISPR-Cas screen data

To illustrate the broader applicability of our method, we used publicly available CRISPR-Cas screen data of [17] (see Additional file 1). Re-scaled but unnormalized data displays considerable variability, both between cell lines, as well as between replicates of the same cell line (Fig. 3d). In addition, some of the guide RNAs suggested as positive controls by the original authors do not yield a lethal phenotype at any time point (Additional file 1: Figure S10). We selected for normalization only guide RNAs that yield a lethal phenotype in at least 50 out of the 57 replicates (see Additional file 1).

As with the siRNA screen data, lethality scores already display empirical distributions more similar across cell lines than raw (scaled) data (Additional file 1: Figure S11 and S12), although considerable variability remains between some of the replicates, notably of colon cancer cell line HCT116_1. In addition, values for replicates of retina epithelial cell line RPE1-TERT (RPE1) have consistently higher counts than for other cell lines (Fig. 3d). Since counts of both library guide RNAs as negative controls are affected, it is clear that this is a technical artefact, rather than a biological difference.

Rscreenorm corrects for artefacts such as increased variability as well as for artificially higher counts. After applying rscreenorm using 95% of the lethality scores as core set (Additional file 1: Figure S13), rscreenorm scores displayed comparable data ranges and spread, while tail differences that represent phenotypic effects (depletion) are preserved (Fig. 3f and Additional file 1: Figure S14). In contrast, median-centering of the data, as for example done by MAGeCK for pre-processing [18], would neither correct the larger variability of HCT116_1 replicates, nor would it correctly center RPE1 replicates (Fig. 3d), suggesting instead that guide RNAs yield more depletion in other cell lines compared with RPE1.

**Fig. 5:**
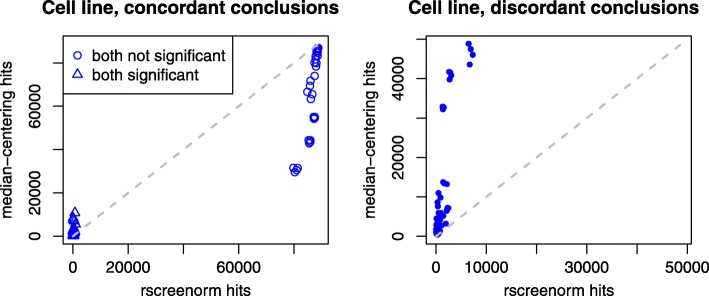
Agreement between hit lists using median-centering or rscreenorm. CRISPR-Cas screen data example, cell line effect: scatterplots of concordance (left) and discordance (right) between lists of selected guide RNAs, with rscreenorm on the x-axis and median-centering on the y-axis

Reproducibility is hampered if artefacts such as the ones identified above are not adequately corrected. We assessed this by comparing test conclusions involving pairs of cell lines with one common cell line per pre-processing method (see Additional file 1). Rscreenorm produced higher agreement fractions between tests that were not significant in both cell line pairs, as well as lower disagreement fractions, when compared to median-centering. In particular, when testing for a cell line effect, lists of guide RNAs that were not significant yielded much higher and less variable overlaps (at least 89%) when using rscreenorm, compared to using median-centering (as low as 33%; Fig. 5, left graph). In addition, the proportion of discordant hits in lists (i.e. those leading to a significant result in one list and a not-significant in another) was much lower (at most 8%) when using rscreenorm, compared with median-centering (as large as 55%; Fig. 5, right graph). Conclusions were similar when examining the time effect, as well as the interaction between time and cel line (Additional file 1: Figure S15). While we found that the use of median-centering yielded a larger number of selected hits, and often those also had larger overlaps than when using rscreenorm, this was achieved at the cost of greatly inflated false positive rates and reduced reproducibility. As the most time- and labour-consuming task in such studies is extensive hit validation, it seems more important to yield a shorter hit list with a truly low proportion of false positives, rather than a longer hit list that likely involves many more false positives.

## Discussion

### Assay controls should be used for normalization

Here we propose rscreenorm, a preprocessing method for independent genetic screens. Rscreenorm makes data from multiple genetic screens comparable by computing a lethality score, representing the phenotype relative to the assay controls, and normalizing their distributions. Importantly, the method preserves different proportions of extreme phenotypes, such as lethal hits, observed between screens.

Just as library features, assay controls typically are affected by technical effects. Since they yield the same phenotype across replicates, they make for ideal pre-processing references. Failure to take assay controls into account during pre-processing may leave uncorrected large parts of data variability (Fig. 2a, d), limiting power to find cell viability effects between screens.

Rscreenorm makes use of the assay controls, in contrast with most methods that ignore technical controls [19]. For example, researchers working with RNAi screen data often simply compute (robust) z-scores, whilst CRISPR-Cas screen data is typically pre-processed as sequencing data [20–22]. In both cases the data functional range, represented by negative and positive controls, is ignored. By using the assay controls, rscreenorm can even reliably correct for plate-specific effects per replicate (Fig. 2). Indeed, having been optimized per experiment, assay controls provide the best references for reliable functional range corrections.

Arguments previously raised against using assay controls for normalization are the lack of universal positive/negative controls that yield consistent phenotypic effects with low variability across cell lines [19] and their frequent location in outer rows/columns of micro-titer plates in arrayed screens that affects performance of controls [23]. Our observations are that assay controls, optimized during screen preparation, represent the extremes of the functional data and are affected by technical effects to the same extent as library features (Fig. 2 and Additional file 1: Figures S5 and S12). As such, assay controls are very useful in making comparable functional ranges. Nevertheless, proper experimental design to ensure controls performance is still important, as also suggested by others [24].

The ideal number of assay controls to be used depends on the type of screen used. For pooled screens, enough controls are needed to estimate median and (possibly) variability per replicate, so a total of about 20 negative and 20 positive controls may be enough. For arrayed screens, plate effect estimation and correction can be reliably done with 4 of each control type per plate, with an absolute minimum of 2. Researchers should also be aware of the fact that, when studying cell depletion via the number of reads per guide RNA, many positive controls yield zero reads. This means in particular that it is possible that all positive controls yield 0 reads for at least one replicate, which corresponds to zero variability. In such cases, core sets can be better constructed by choosing a proportion of library lethality scores, rather than using the distance between controls.

### Comparison with classic quantile normalization and z-scores

Classic quantile normalization centers replicates around the same value, as do z-scores, without taking assay controls into account. Such centering can thus not guarantee that resulting values represent similar phenotypes across screens. In particular, in cases where biological replicates under study display widely different susceptibilities to gene perturbations, screen data for different biological replicates typically display shifts associated with the effects under study. This is often the case when different cell lines and treatment conditions are studied. Contrary to what is desired, simple centering would correct this effect away.

Then why not applying classic quantile normalization to the lethality scores? Firstly, computing the lethality scores is essential, as this makes functional ranges across replicates comparable. In particular, similar values observed for different screens will then represent similar phenotypes, as was shown in the “Results” section. Secondly, classic quantile normalization would make lethality scores distributions the same across replicates, over the entire range of measurements. In particular, it would make the tail empirical probabilities *P*{*Z*_*ik*_>*z*} equal to *p*(*z*) across all replicates *k*=1,…,*K*. In other words, the proportion of extreme lethality scores, representing lethal phenotypes, becomes the same across replicates.

This is undesirable, since even untreated cell lines may display different sensitivity to genetic interference, yielding different proportions of lethality-yielding features (see “Results” section). In studies involving different conditions, such as the effect of genetic interference with and without treatment, proportions of lethal features may differ even more than between cell lines under the same conditions. Thus, classic quantile normalization applied to the entire range of phenotypes would be likely to remove effects one wishes to find, as we showed in the simulation study.

In the siRNA screen literature, many works made use of (robust) z-scores to make data from different screens comparable. This may intuitively seem an adequate step, since z-scores have mean 0 and variance 1 for each replicate. However, in addition to yielding values that may not correspond to similar phenotypes, empirical data distributions may still differ even if variances are the same. Finally, this ignores important technical effects such as those caused by different plates (see siRNA screen data example below). We conclude that z-scores leave much of undesirable noise intact in data and is, thus, not a good method to make data from multiple screens comparable.

### Reference sets

Some authors have suggested to use an empirical set of features [17] as a reference. This has at least two drawbacks; firstly, such a set of features is constructed in independent studies and therefore are not specifically designed to capture technical variation arising in a new study. Secondly, genetic screens are also applied to different cell lines under different experimental conditions, and it is unclear if such empirical sets of features would remain relevant.

The use of a reference set of features for normalization was previously proposed by others [25, 26] in the context of microarray data normalization, where the reference set represented the entire range of intensities. In contrast, genetic screens may involve different cell lines under different experimental conditions, yielding different proportions of phenotypic effects across samples. Therefore we not only avoid using a core set of features by using a core set of values instead, but also only make the distributions within the core sets the same, thus avoiding over-correction of lethal effects. Indeed, rscreenorm lets the fraction of features with extreme phenotypic effects vary between screens. Our results show that this approach works well to make both data distributions more similar (Fig. 3) and data values between genetic screens comparable (right graph in Additional file 1: Figure S8), as well as to improve reproducibility of results.

### Reducing false positives

In our simulation studies, rscreenorm data yielded false positive rates within the expected ranges, even when positive controls were biased. In contrast, not-normalized data yielded much larger false positive rates than expected. This may be seen by some researchers as a relatively small drawback, given that not-normalized data also yielded more true positives than rscreenorm in most situations. However, it is not difficult to find a method that gives more true positives than another, if false positives can be disregarded. Indeed, a selection yielding all features under study will always yield 100% true positives, and requires no statistics at all. On the other hand, methods that simply yield a false positive proportion below a required level are not necessarily desired: if no features at all are selected, there are zero false positives, but the result is useless. The difficult task is to find true positives, whilst at the same time keeping false positive rates under control. This is precisely what rscreenorm does.

Yielding acceptable true positive rates with expected false positive rates is also important for follow-up experiments. Subsequent validation experiments are often time consuming and labour intensive and, as such, benefit from short enough hit lists with known false positive proportions. Rscreenorm was shown to preserve false positive rates across a range of control levels, so if longer hit lists are desired, researchers need only change the FDR cut-off.

### Applicability

Rscreenorm is non-parametric and may make use of robust statistics. It can thus be applied to studies with a wide range of designs, as long as assay controls that define the extreme phenotypes are present, and a representative proportion of data from each replicate can be used to define core set values. In particular, smaller screens composed of hits found in previous studies should also include features that were found not to change between the experimental conditions under study. Such features should reliably represent the range of values representing common phenotypes, to enable adequate normalization of values and reliable downstream analyses. While researchers may feel the inclusion of non-hits in a screen is a waste of resources, in light of our analyses a much bigger waste would ensue if adequate normalization cannot be done, producing too many false positives.

### Reproducibility

One of the biggest problems currently with analysis of genetic screens data is the low reproducibility of hits in validation screens and the limited overlap between independent studies [2, 10–12]. Using both simulations and experimental data, we showed that rscreenorm can not only increase the proportion of true positives and improve correlations between screens of different studies, but also increase results’ reproducibility.

## Conclusions

Rscreenorm successfully normalizes data from multiple genetic screens by taking the functional nature of the data into account. This corrects for undesired variability while making phenotypes be represented by similar values across replicates of all screens. It keeps the proportion of false positives in hit lists under control, and improves reproducibility between different studies.

## Additional file

Additional file 1 Supplementary materials. (PDF 3186 kb)

## Abbreviations

FDR: False discovery rate
gRNA: Guide RNA
RNAi: RNA interference
RPE1: Retina epithelial cell line RPE1-TERT siRNA: Small-interference RNA
