# Supplementary material for: Rscreenorm: normalization of CRISPR and siRNA screen data for more reproducible hit selection
Source: BMC Bioinformatics. 2018 Aug 20;19:301. doi: 10.1186/s12859-018-2306-z (PMC6102854; doi:10.1186/s12859-018-2306-z)
Supplement: Supplementary file 1 — Supplementary materials. (PDF 3186 kb) [file 12859_2018_2306_MOESM1_ESM.pdf]

Rscreenorm: normalization of multiple CRISPR and siRNA screens for more reproducible hit selection
Supplementary Material

Costa Bachas
Jasmina Hodzic
J. Cornelis van der Mijn
Chantal Stoepker
Henk M. Verheul
Rob Wolthuis
Emanuela Felley-Bosco
Wessel van Wieringen
Victor W. van Beusechem
Ruud Brakenhoff
Renée X. de Menezes

July 19, 2018

Contents

1 Core set of lethality scores **3**
1.1 Alternative definition 3
1.1.1 Core set definition using assay controls 3
1.1.2 Interpretation of the cut-off 4
1.1.3 Choosing γ 4
1.2 Studying depletion as well as proliferation effects 5

2 Simulation study **5**
2.1 Motivation 5
2.2 No cell line effect 6
2.3 Cell line effect 6
2.4 Biased positive conttrols 7

3 Cell lines **8**
3.1 Cells and conditions 8
3.2 siRNA screens 9
3.3 CRISPR-Cas screens 9
3.4 Supplementary table: cell lines 9

4 Example: siRNA data sets **11**
4.1 Studies involved 11
4.2 Reading in and scaling viability data 11
4.3 Negative controls comparable 12
4.4 Core sets for the siRNA screens 12

5 Example: CRISPR-Cas screen data **13**
5.1 Data set used 13
5.2 Count data often has zeroes 13
5.3 Lethality scores and quantile normalization 14
5.4 Assessing reproducibility 15

6 Supplementary figures **16**

1 Core set of lethality scores

1.1 Alternative definition

1.1.1 Core set definition using assay controls

The core set is defined as the set of lethality scores forming the core of the scores' distribution, per replicate. As such, it should be formed by a part of the scores' distribution that can be expected to be the same across replicates and cell lines, corresponding to phenotypes that are observed across all screens in similar proportions. In the main text we built it by choosing a fixed percentage of all lethality scores. This seems a reasonable choice in studies where educated guesses can be made about the proportion of viabilities overlapping between different screens, such as when whole-genome screens are used. However, in dedicated screens using a subset of all library features, it may be harder to choose such a proportion. As an alternative, we propose to define the core set using their distances relative to the assay negative and positive controls. Since the core set is meant to exclude extreme (lethal) phenotypes, we will define it as the set of values closer to the negative controls than to the positive controls.

Specifically, per replicate k we defined distances from each lethality score to the negative controls distribution as:

$$d_{ik}(Z^N) = \frac{|Z_{ik} - \tilde{Z}_k^N|}{\text{MAD}_k^N}, \text{ for replicate } k,$$

where Z_{ik} represents the (re-scaled) observed value for feature i of replicate k, $\tilde{Z}_k^N$ and MAD_k^N represent the median and median absolute deviation (MAD) respectively of lethality scores for negative controls and $|x|$ represents the absolute value of x. A similar expression was used to yield distances from each lethality score and those of the positive controls, producing $d_{ik}(Z^P)$. Then, for each replicate k, the core set $\{Z_{ik}^{\text{c}}\}$ is formed by lethality scores $\{Z_{ik}, i = 1, \ldots, C_k\}$ such that $\gamma d_{ik}(Z^P) > d_{ik}(Z^N)$, for some fixed $\gamma > 0$. Note that the number C_k of scores included in $\{Z_{ik}^{\text{c}}\}$ typically varies with the replicate k. Since the distance used was standardized by the controls' variability, it takes technical variability into account.

In the distance defined above, we standardize the difference between each lethality score and the center of the controls' distribution by a measure of the controls' variability. We suggested using the MAD as it is a robust measure. However, in case the data at hand is obtained via deep sequencing, such as is the case with pooled gene-silencing screens, this may be undesirable. Indeed, positive controls may then involve many zeros, yielding possibly MAD=0 for some replicates. Note that it is often not the case that positive controls have indeed zero variability, so this is just an artefact. In such cases, alternatives for the MAD are the inter-quartile range, which is slightly less robust, but even it may yield zeros, or the classic standard deviation.

1.1.2 Interpretation of the cut-off

We proposed to use a distance defined in terms of the scaled difference to the center of each control distribution, standardized by its median absolute deviation. This implies that, for a feature with lethality score z and $\gamma = 1$, the distances are $|z - \tilde{Z}^N|/\text{MAD}_{Z^N}/$ and $|z - \tilde{Z}^P|/\text{MAD}_{Z^P}$, where all values refer to the same replicate but the index k is omited for clarity. If we note that the lethality scores for negative controls are centered at 0 and those for positive controls are centered at 1, we can re-write these distances as $|z|/\text{MAD}_{Z^N}$ and $|z - 1|/\text{MAD}_{Z^P}$. So, the cut-off for the core set is the value z_c such that these distances are equal, which means z_c must satisfy

$$\frac{|z_c|}{\text{MAD}_{Z^N}} = \frac{|z_c - 1|}{\text{MAD}_{Z^P}}$$
$$\frac{|z_c|\text{MAD}_{Z^P} - |z_c - 1|\text{MAD}_{Z^N}}{\text{MAD}_{Z^N}\text{MAD}_{Z^P}} = 0.$$

Since the cut-off z_c is by definition between the medians of negative and positive controls, clearly it satisfies $0 < z_c < 1$, so we can write:

$$\frac{z_c\text{MAD}_{Z^P} + (z_c - 1)\text{MAD}_{Z^N}}{\text{MAD}_{Z^N}\text{MAD}_{Z^P}} = 0$$
$$\frac{z_c(\text{MAD}_{Z^P} + \text{MAD}_{Z^N}) - \text{MAD}_{Z^N}}{\text{MAD}_{Z^N}\text{MAD}_{Z^P}} = 0$$
$$z_c(\text{MAD}_{Z^P} + \text{MAD}_{Z^N}) = \text{MAD}_{Z^N}$$
$$z_c = \frac{\text{MAD}_{Z^N}}{(\text{MAD}_{Z^P} + \text{MAD}_{Z^N})}.$$

So, the cut-off is essentially set as a relationship between the controls' variabilities.

Specifically, if the negative controls have very small variability compared with the positive controls, the cut-off will be very small. If in addition the library features display (considerably) more variability than the negative controls, then lethality scores selected by this distance will not represent well the core of the scores distribution.

1.1.3 Choosing γ

We suggest choosing γ so that a large enough percentage of lethality scores is included in the core set. Specifically, we consider choosing the cut-off so as to guarantee a certain representation of all lethality scores or, in other words, to guarantee a given proportion of lethality scores is included in the core set. The specific proportion may vary according to the fraction of siRNAs believed to yield a different lethality status across cell lines. In genome-wide screens this fraction may lie between 0.5% and 5%, but this may also vary across cell lines.

Assuming at most 5% of scores differ in their lethality status, we can use the 95th percentile $Z_{.95}$ of the scores as the cut-off z_c to set γ, as in:

$$Z_{.95} = \gamma \frac{\text{MAD}_{Z^N}}{(\text{MAD}_{Z^P} + \text{MAD}_{Z^N})} \Rightarrow \gamma = \frac{Z_{.95}(\text{MAD}_{Z^P} + \text{MAD}_{Z^N})}{\text{MAD}_{Z^N}}. \quad (1)$$

Alternatively, in studies where a large proportion of library features is expected to display a lethal phenotype, a value of γ such as 1, halfway between negative and positive controls, may be more appropriate. The specific γ value used will then determine the part of the lethality scores' distribution that will be normalized to being the same across replicates and cell lines.

1.2 Studying depletion as well as proliferation effects

Some studies are designed to measure both depletion as proliferation effects – in other words, both lethal as well as growth-promoting features are of interest. In such cases, the core set should include the core of the lethality scores' distribution excluding both tails. One relatively simple way of doing this is to define each replicate's core set as all values between the 2.5 and the 97.5 percentiles, for example, leaving 2.5% of values on each tail.

Alternatively, distances from negative and positive controls distributions can be used, for example by including in the core set all lethality scores in a symmetric interval around z_c, such as $[z_c - \delta, z_c + \delta]$ for some $\delta > 0$, where z_c is the score value satisfying $d_{ck}(Z^N) = d_{ck}(Z^P)$, as defined in subsection 1.1.2.

2 Simulation study

2.1 Motivation

Here we assume that 6 cell lines are available, each being screened in triplicate. Per replicate, lethality score measurements are obtained for a number ($M = 1000$) of library features are studied, as well as for negative and positive controls (200 each). Interest lies in finding library features that yield different phenotypes between cell lines. In this context, scores are negatively associated with viability, with higher values representing less viability.

All entries in the data matrix are drawn from independent normal distributions, with parameters varying as follows. Controls' lethality scores are drawn with standard deviation 0.1; negative controls' lethality scores have mean 0 whilst positive controls have mean 1. Library features are drawn from a normal distribution with standard deviation twice that of controls, each with a different mean $\mu_i (i = 1, \ldots, 1000)$ that itself is drawn from a beta distribution. In order to introduce heterogeneity between the library features, we generate means assuming $\mu_i \sim \mathcal{B}(2, 6)$, so that the beta distribution is asymmetric to the left, with expected value $2/8 = 0.25$, so that most library features yield little lethality. The above sets parameters under H_0, which states that there are no library features with differential phenotypes between cell lines.

To make the data more realistic, we also introduce a stretch/contraction effect, which consists in multiplying the signal of library lethality scores as well as positive controls' mean by a fixed value, per replicate. Constants used here are: 0.7 (cell lines 1, 4) and 1.4 (cell lines 3, 6). Data for cell lines 2, 5 was neither stretched nor contracted after simulation.

2.2 No cell line effect

Data generated showed enough separation between controls distributions, as well as the signal stretching/contraction expected (see top-left column graph in supplementary figure 1). When overlayed, however, these signal range changes could seem to suggest a cell line effect, even though no true cell line effect is present in this case and the signal stretching/contraction is orthogonal to the tested cell line effect.

The generated data is then normalized as follows. For each replicate, we define the core set as all lethality scores up to the 95th percentile. These values are then quantile-normalized, and the same normalization is extended to the remainder of the data. This corrects for the signal range changes in the appropriate way (see top-middle column graph in supplementary figure 1).

Small differences on the densities' tails may remain after rscreenorm, but these do not lead to false positives. To illustrate that, we fit a linear regression model per library feature, where we test for a non-existent group effect: group 1 includes cell lines 1, 2 and 3, whilst group 2 includes the remaining cell lines. Note that this group effect is orthogonal to the stretch effect. The p-values for this comparison obtained with the quantile-normalized data seem to follow a uniform distribution, which would be expected under no effect (data not shown). In contrast, p-values for the same comparison obtained using the lethality scores show an enrichment of small values, suggesting that there is an effect when the data does not (data not shown). Indeed, corresponding false discovery rates (bottom-left graph in supplementary figure 1) yielded when using the un-normalized lethality scores are well above the expected value, for all FDR-control levels used. In contrast, when using the rscreenorm data false discovery rates remained around the expected value.

2.3 Cell line effect

In order to generate differential effects for library features, we assume that 80% of all library features is not affected and thus has observations generated as described above. For the remaining 200 library features, we generate observations for half of the cell lines (1, 2 and 3) in the same way, whilst the other half is generated with a mean of 0.5.

Generated lethality scores for library features had distributions that varied considerably (bottom-left graph in supplementary figure 1). After rscreenorm, for which 95% of the data for cell lines 1, 2, 3 and 70% of that for cell lines 4, 5 and 6 were used, the data distributions were much more similar (bottom-middle graph in supplementary figure 1). Here we point out that the upper tail

of distributions differ, allowing for different proportions of scores being extreme. Of course, in practice the proportion of of signal that overlaps is unknown, so researchers may avoid the arbitrariness of a set-proportion and instead build core sets using distances relative to controls, as described in subsection 1.1.1.

In order to better understand the impact of using rscreenorm in this case, we simulate 1000 datasets in the same way described above. We then again fit a linear regression model including a group effect (cell lines 4, 5, 6 *vs.* 1, 2, 3) per library feature. Un-normalized lethality scores consistently yield false positives, in agreement with what was observed for the case with no effect. For features with effect between the two cell lines groups, un-normalized lethality scores results yield somewhat more power, at the cost of more false positives. This is confirmed by the corresponding false discovery rates (bottom-right graph in supplementary figure 1), where we can see that the un-normalized lethality scores often yielded proportions above the expected value, given by the FDR-control level used. In contrast, when using the rscreenorm data false discovery rates remained around the expected value.

2.4 Biased positive conttrols

The setups so far described assume that both negative and positive controls yield responses in accordance to their phenotypes. In practice, however, assay controls can be biased, for example due to unforeseen technical issues. A bias on the negative controls could be circumvented by centering the screens around the median response of library features, say, since it is robust to features with lethal phenotype. However, a bias on the positive controls is harder to correct for, unless there are enough known library features with lethal phenotype, which is not always the case.

To evaluate the effect of positive controls bias on results, we extend the simulation study above to include such a bias. Specifically, we will introduce biases as follows: for cell lines 1, 2, 3, we add respectively $-0.2, 0, 0.2$ to the positive controls means, and subtract the same values from the library features means. The same is done for cell lines 4, 5, 6, so that cell lines in each group are affected in the same way. Since the stretch effect is a multiplicative effect with mean $0.6, 1, 1.15$ for cell lines 1, 2, 3 of group 1 respectively, and cell lines 4, 5, 6 of group 2, the combination of bias and stretch leads to positive control means, which were originally equal to 1, to be approximately

group 1	0.4	1	1.35
group 2	0.4	1	1.35,

where we point out that this is taken as the mean across each cell line's replicates, and a normal error is added with standard deviation 0.1 to yield per-replicate positive control means. Note also that the positive controls variability is kept unchanged, whilst library features will vary more or less depending on the stretch. In this case, we see that, for cell lines 1 and 4, the positive control

means are slightly smaller than that for library features. We will refer to this bias setting as “bias 1”.

We will also consider a second situation where, for cell lines 1, 2, 3, we add respectively $0.2, 0, -0.2$ to the positive controls means, and subtract the same values from the library features means. The same is done for cell lines 4, 5, 6, as before. In this case, the combination of bias and stretch leads to positive control means to be approximately

$$\begin{array}{llll} \text{group 1} & 0.8 & 1 & 0.95 \\ \text{group 2} & 0.8 & 1 & 0.95, \end{array}$$

where, as before, a normal error is added with standard deviation 0.1 to yield per-replicate positive control means. In this case, we see that, for cell lines 1, 3, 4 and 6, the positive control means are smaller than 1. We will refer to this bias setting as “bias 2”.

These different setups are illustrated by the corresponding distributions of controls, as well as for the library feature means, in supplementary figure 3. Note that the main difference between the no-bias and bias 1 situations are for cell lines 1, 4, which display larger lethality scores in bias 1 than without any bias. On the other hand, in bias 2 library features for cell lines 1, 4 overlap less with positive controls than without any bias.

Subsequently, 1000 independent datasets are simulated under each bias type, and both false positive rates as true positive rates yielded by the analysis as before are evaluated (supplementary figure 4). Interestingly, in the bias 1 situation, when rscreenorm is used more true positives are yielded than when no normalization is used, which contrasts with what is observed for the no-bias situation. This seems to follow from the higher lethality scores for cell lines 1 and 4 in bias 1, and rscreenorm correctly separates the group effect from the bias. In the bias 2 situation, rscreenorm yields less true positives than no normalization, similarly to the no-bias situation. Here we should point out that, in all cases, rscreenorm results yield false positive rates within the expected range, whilst the data without normalization yields (much) higher false positive rates.

3 Cell lines

3.1 Cells and conditions

A549 and SW1573 non-small cell lung cancer (NSCLC) cell lines, VU-SCC-120 and VU-SCC-1131 head and neck squamous cell carcinoma (HNSCC) cell lines, PC-3 prostate cancer (PCa) cell line and 786-O renal cancer (RCC) and SV40 immortalized primary fibroblast cell line VU-1149 were cultured using the appropriate culture medium with glutamine and penicillin/streptomycin added, in a humidified atmosphere of 5% CO_2 at 37°C (conditions listed in Supplementary Table I).

3.2 siRNA screens

Cell lines were subjected to high-throughput forward or reverse transfection in 272 96-wells or 68 384-wells plates. Experimental conditions were optimized to achieve amongst others optimal cell densities, optimal concentrations of transfection reagents, and the most suitable assay duration. In replicates of 21,121 wells, genes were targeted in duplicate (96-wells) or triplicate (384-wells) using SMARTpool siRNAs of the siARRAY Human Genome library (Dharmacon, part of GE Healthcare, LaFayette, CA, Cat. No. #G-003500, #G-003600, #G-004600 and #G-005000). The siRNA pools were transfected into the cells using DharmaFECT1 (Dharmacon) or RNAiMax (Thermo Fisher Scientific,Waltham, MA, USA) transfection reagent. The non-targeting control siRNA siControl#1 or siControl#2 used as negative control (siCon; Cat. No. #D-001210-01-05 and #D-001210-02-05, Dharmacon) and siRNA targeting the polo-like kinase 1 gene (siPLK1; Cat. No. #M-003290-01, Dharmacon) as a positive control. Note that the library also contains siPLK1; we will refer to the former as positive controls and to the latter as sample siPLK1. The siRNAs are identical but the positive controls siRNAs were not part of the library and added manually to the plates. After transfection, cells were cultured for 4-7 days. Subsequently, cell viability was assessed either using the Cell-Titer Blue® assay (CTB; Promega Benelux, Leiden, The Netherlands) or by counting of nuclei using 4',6-diamidino-2-phenylindole (DAPI) or Hoechst 33342 automated cytometry readout on an Acumen eX3 plate reader (TTP Labtech, Melbourn, United Kingdom).

3.3 CRISPR-Cas screens

We also apply our method to the publicly available CRISPR-Cas screening data of Hart et al. [18]. We obtained the read count data for 9 cell lines that were screened using the basic library of roughly 90K gRNA from http://tko.ccbr.utoronto.ca/. The library contains gRNAs that are cloned in lentiviral vectors, pooled and introduced in the cells simultaneously with genome-wide coverage. After selection and a certain incubation time, gRNAs that are depleted from the pool at later time points versus the T_0 control are identified by next generation sequencing. Absence of a particular gRNA is an indication that the gene targeted by that guide RNA is essential for that cell line.

3.4 Supplementary table: cell lines

Cell line name	Cell type	No. repl.	Plate type ‡	Culture medium / % FCS	Transfection type	Conc † (nM)	Transfection reagent	Duration ‡ (h)	Doubling time (h)	Viability Assay	Negative control	Positive control
A549	NSCLC	2	96	DMEM/5	forward §	25	DharmaFECT1	120	22	CTB	siCon#1	siPLK1
SW1573	NSCLC	2	96	DMEM/5	forward §	25	DharmaFECT1	96	22§	NC *	siCon#2	siPLK1
VU-SCC-120	HSNCC	2	96	DMEM/5	forward §	25	DharmaFECT1	96	20	CTB	siCon#2	siPLK1
VU-SCC-1131	HNSCC	3	384	DMEM/5	reverse	25	RNAiMax	120	24	CTB	siCon#2	siPLK1
VU-1149	Fib	3	384	DMEM/10	reverse	25	RNAiMax	96	NA	CTB	siCon#2	siPLK1
PC-3	PCa	2	96	RPMI1640/10	forward §	20	DharmaFECT1	168	25-50 §	NC *	siCon#2	siPLK1
786-O	RCC	3	384	DMEM/10	reverse	20	DharmaFECT1	72	22-45 &	NC *	siCon#1	siPLK1

Table 1: Summary of experimental conditions for 7 human genome wide siRNA screens. † siRNA concentration; ‡ Assay duration after transfection; NSCLC = non-small cell lung cancer; PCa = prostate cancer; Fib = Simian virus 40 immortalized fibroblasts; HNSCC = head and neck squamous cell carcinoma; RCC = renal cell cancer; DMEM = Dulbeccos Modified Eagles Medium (Lonza, Verviers, Belgium); FCS = Fetal Calf Serum; *source Nagel et al; § source NCI, ATTC, DSMZ; * NC = Nuclei count; & source NCI, Williams et al, Cowley et al; CTB = CellTiter-Blue assay; siCon = negative control siRNA pool; siPLK1 = smart pool siRNA targetting PLK1 Polo-like kinase 1 mRNA; § for forward transfections, cells were seeded 24 hours prior to transfection.

4 Example: siRNA data sets

4.1 Studies involved

To illustrate our method, we will consider data from whole-genome siRNA screens corresponding to multiple cell lines. Here we use data obtained from the following studies:

- Sanne Martens-de Kemp (head & neck cancer, published)
- Jasmina Hodzic (prostate cancer, unpublished)
- Koen van der Mijn (renal cancer, unpublished)
- Remco Nagel (lung cancer, published)
- Chantal Stoepker (head & neck cancer, unpublished)
- Ilya Kotov (lung cancer, published)
- Job de Lange (fibroblasts, published)

Each one of these studies has performed siRNA screens, using an experimental protocol that suited the specific cell line used, such as incubation time, and the study objectives. All studies involved measuring cell viability for the whole genome using the same Dharmacon siRNA library, distributed either over 272 96-well plates, as was the case in the studies of Martens, Hodzic, Kotov and Nagel, or over 68 384-well plates, as in the studies of van der Mijn, Stoepker and de Lange. Each study involved a single cell line under at least two conditions, one of which was untreated or wild type. To illustrate our method, only the untreated/wild type replicates of each study were used.

The negative control used in each study is typically a siControl. The positive control used was in all cases *siPLK1*. Plate design involved controls on all plates, positioned on the second column of the plate, in cases where 96-well plates were used, or on the first four columns, in cases where 384-well plates were used. The number of controls per plate may vary per experiment.

The read-out method also varied per experiment. The Hodzic, van der Mijn and Nagel studies used automated cell counting after fluorescent nulcear staining, whilst the remaining used whole-well metabolic activity (CTB conversion) measurement.

4.2 Reading in and scaling viability data

Data for each replicate was read into R[R Core Team, 2016], configured and annotated using the package cellHTS2[Boutros et al., 2006] and gene annotation files. Although different studies used different plate sizes, the whole-genome siRNA library used was the same. Thus, after configuration and annotation, the same siRNAs had measurements for all experiments. As plate design did differ across studies, the number of controls varied.

After configuration, all viability values were log2-transformed, to introduce some regularization to the data. Although this is not necessary for our non-parametric pre-processing method to work, which is also robust to extreme values, we here used this transformation to yield better visualization.

No spatial trends were observed for specific plates, so pre-processing progressed by investigating other technical effects.

4.3 Negative controls comparable

We first centered data per replicate around its negative controls, which in essence yield the same phenotype across the entire data. We then estimate technical effects using the negative controls, and subsequently correct the entire data. Technical effects that can be corrected in this way include plate and screening date effect.

Specifically, we fitted a regression model to the log2-transformed viability value for all sample siRNAs. The model included design-specific effects such as plate and seeding day, for studies where this was needed. The negative control-centered viability data X^*_{ijk} can be interpreted as the (scaled) viability for siRNA i relative to the average negative control viability.

While the data is now centered around the same value, the functional ranges varies, represented by values between negative and positive controls. Lethality scores fix this, yielding comparable functional ranges between replicates and replicates.

Lethality scores still vary due to both technical factors, as illustrated by the controls' distributions, as well as biological ones. We noted in particular that library siRNAs' lethality scores distributions varied across cell lines and replicates more than controls, illustrating variability arising from both sources. In contrast with controls that were spotted on plates by hand, library siRNAs were robot-spotted on plates, so their viability values (and lethality scores) should yield very comparable distributions across replicates. While differences on the upper tail of distributions could be attributed to varying proportions of lethal siRNAs across cell lines, the core of the distributions can be safely assumed to be the same – differences arising typically due to factors that are not directly related to phenotype and, thus, not of interest. So, we will quantile normalize each replicate's lethality scores to make the core of their distributions the same.

4.4 Core sets for the siRNA screens

It is important that this core set includes enough of the lethality score values, so as to represent well the core of its distribution. In the current study, we started off by setting $\gamma = 1$ for all replicates for consistency. However, we noted that the core sets' representation of the lethality scores distributions was relatively low for replicates of the studies using PC-3 and the A549 (supplementary figure 7). Indeed, the core set fell too short of the tail, due to the relatively small negative control variances for these replicates.

Each distribution reflects biological and technical variability between siRNAs, typically with no or low lethality, and they vary due to experimental variability. However, if conditions could have been kept precisely the same, we would have expected these distributions to also be the same. Thus, it is reasonable to assume that the core set's lethality scores distributions are the same for all replicates, per experiment.

Our quantile normalization not only minimized variability between replicates within the same experiment, but also made lethality scores between experiments more comparable (bottom graphs in figure 4 of the main text). Here it is obvious to see that the empirical distributions mostly overlapped in the low-lethality range, the part of the distribution actually normalized. Differences on the upper-tail remained, as they should, preserving phenotypical information.

Another way to illustrate this improvement is to compute the correlations between replicates before and after normalization. The density plot of all pairwise correlations between replicates shows that our pre-processing approach essentially corrected effects that increase the lowest correlations (left graph in supplementary figure 8). Considering now the individual correlations, we noticed indeed that most change very little (less than 0.05), except for those involving A549 replicates: of those involving the first replicate, three decreased by more than 0.05, whilst of those involving the second replicate, almost all increased by more than 0.05 (right graph in supplementary figure 8). These corresponded indeed to the low-correlations shoulder that improved in the density plot. So our normalization has the potential to improve correlations between replicates, where needed.

5 Example: CRISPR-Cas screen data

5.1 Data set used

Being non-parametric, rscreenorm can also be used to pre-process count data with a large number of zeroes, such as pooled CRISPR-Cas screen data arising from multiple cell lines. We illustrate this by applying it to a publicly available CRISPR-Cas screen data set, published and previously analysed by Hart et al. [2015], and available via the url `http://tko.ccbr.utoronto.ca/`. Specifically, the data refers to five different cell lines, each screened once at $T = 0$ and subsequently at multiple time points, chosen according to the cell line-specific duplication time. We used the read counts of the basic library containing roughly 90k guide RNAs. At $T = 0$ a single replicate was obtained, whilst for later time points at least duplicates were generated. A total of 57 screens were produced, with the number of replicates per cell line given in supplementary table 2.

5.2 Count data often has zeroes

The tab-delimited files with data for the various cell lines were read in, together with guide RNA ids as well as control ids. This being count data, there may

Time<br>Cell line	T_0	$T > 0$
DLD	1	3
GBM	1	6
HCT116.1	1	10
HCT116.2	1	12
HeLa	1	12
RPE1	1	8

Table 2: Number of replicates available from the TKO data, per cell line and time point.

be measurements precisely equal to zero. While zeros are widespread in the data, they are informative because of their relationship with the phenotype (depletion), and thus cannot be disregarded. As data transformation, we used the hyperbolic arc sine, that keeps measurements equal to zero with the same value, while transforming the data scale. It has been shown [Tibshirani, 1988] to be the transformation that corrects as much as possible for the dependence of the variance upon the mean. Although this step is not necessary for our non-parametric pre-processing method to work, it yields better visualization.

After transforming the data, densities of the values per replicate and per guide RNA type (library, negative and positive control) were made (figure 1 of the main text). These densities show that there is considerable variability between screens for different replicates and cell lines, in particular with measurements at $T > 0$ displaying a shift compared to those for $T = 0$.

5.3 Lethality scores and quantile normalization

We took 778 guide RNAs labelled as "chr10Promiscuous" for positive controls, as these were most likely to yield depletion according to Hart et al. [2015]. These guide RNAs had a bimodal distribution of values per replicate, with the left-most peak seemingly corresponding to guide RNAs leading to depletion, whilst the right-most peak corresponding to a mild phenotype (data not shown). We decided to use as positive controls the subset of chr10Promiscuous guide RNAs that yielded values as often as possible under the left-most peak. By using a cut-off between the two peaks as $c = 4$ for replicates observed at $T = 0$, and as $c = 2$ for replicates observed at later time points, we could separated observations between two peaks for all replicates. A total of 224 chr10Promiscuous guide RNAs with distinct sequences had their values below c for at least 50 cases, out of the total 57 observed. This formed the set of positive controls used hereafter.

The next step was to define core sets, and use those to quantile-normalize the data. We used the same distance definitions as before, where the median and MAD per control type and replicate are used for negative controls. However,

due to the large number of (informative) zeroes in the data in general, and in particular in the positive controls, their MADs often yield zero. This was still the case for the slightly less robust inter-quartile range (IQR), so we decided to use the standard deviation as variability measurement in the computed distances.

The negative controls' lethality scores yielded similar spread to that of library guide RNAs, so after setting a value $\gamma = 1$, the majority of score values were included in the core sets (data not shown). Importantly, the right tails of the library guide RNAs distributions were not included in the core sets. Per replicate, at least 90% of all scores were included in the core set, which was considered acceptable.

Subsequently, quantile normalization was applied. As expected, the normalized data for the various cell lines, time points and replicates displayed a similar distribution, except for the tail where differential phenotype may still be detected (figure 1 in the main text). Importantly, after normalization the variability of measurements per guide RNA decreased substantially (data not shown).

We conclude that our pre-processing method can be used on CRISPR-Cas screen data to correct for differences between replicates, and that it helps to decrease technical variability in the data.

5.4 Assessing reproducibility

We would like to assess the reproducibility of results obtained with rscreenorm, and compare these to results obtained after median-centering the data. The latter is the pre-processing done by MAGeck (Li et al. [2014]). In order to assess reproducibility, we fit a regression model to replicates corresponding to each pair of cell lines, involving a cell line effect, a time ($T = 0$ vs $T > 0$) effect and an interaction between these two factors. P-values are extracted for each effect as well as for the interaction. Cell line pairs with one cell line in common are likely to yield an overlap between gRNAs found to have an effect, either because they are cell line-specific, or because they are lethal for both cell lines. So, we compare hit lists between pairs having one cell line in common by producing tables of these test results. This is done for hit lists yielded for three different cut-offs selecting hits: $p \leq 0.01$, $p \leq 0.001$ and $p \leq 0.0001$.

Each table produces two counts for concordance between test conclusions (either both tests are not significant or both tests are significant), as well as two counts for discordance. Concordance and discordance counts can now be compared between those yielded by rscreenorm and those yielded by median-centering. Scatterplots of these counts are in supplementary figure 8. From this figure, we can see that concordance counts are typically higher for rscreenorm when both results are not significant, and discordance results are typically lower with rscreenorm, compared with median-centering. Concordance counts are sometimes slightly higher with median-centering than with rscreenorm. Interestingly, this trend is the same regardless of the effect considered (cell line, time and the interaction between them). This suggests that concordance/discordance counts are data-led, rather than linked to a biological effect.

We conclude that concordance is mostly higher, and discordance is almost always lower, with rscreenorm compared with median-centering.

6 Supplementary figures

References

M Boutros, LP Bras, and W Huber. Analysis of cell-based rnai screens. *Genome Biology*, 7(7):R66, 2006.

T Hart, M Chandrashekhar, M Aregger, Z Steinhart, KR Brown, G MacLeod, M Mis, M Zimmermann, A Fradet-Turcotte, S Sun, P Mero, P Dirks, S Sidhu, FP Roth, OS Rissland, D Durocher, S Angers, and J Moffat. High-resolution crispr screens reveal fitness genes and genotype-specific cancer liabilities. *Cell*, 163:1515?1526, 2015.

W Li, H Xu, T Xiao, L Cong, MI Love, F Zhang, RA Irizarry, JS Liu, M Brown, and XS Liu. Mageck enables robust identification of essential genes from genome-scale crispr/cas9 knockout screens. *Genome Biology*, 15:554, 2014.

R Core Team. *R: A Language and Environment for Statistical Computing*. R Foundation for Statistical Computing, Vienna, Austria, 2016. URL `https://www.R-project.org/`.

Robert Tibshirani. Estimating transformations for regression via additivity and variance stabilization. *Journal of the American Statistical Association*, 83 (402):394–405, 1988.

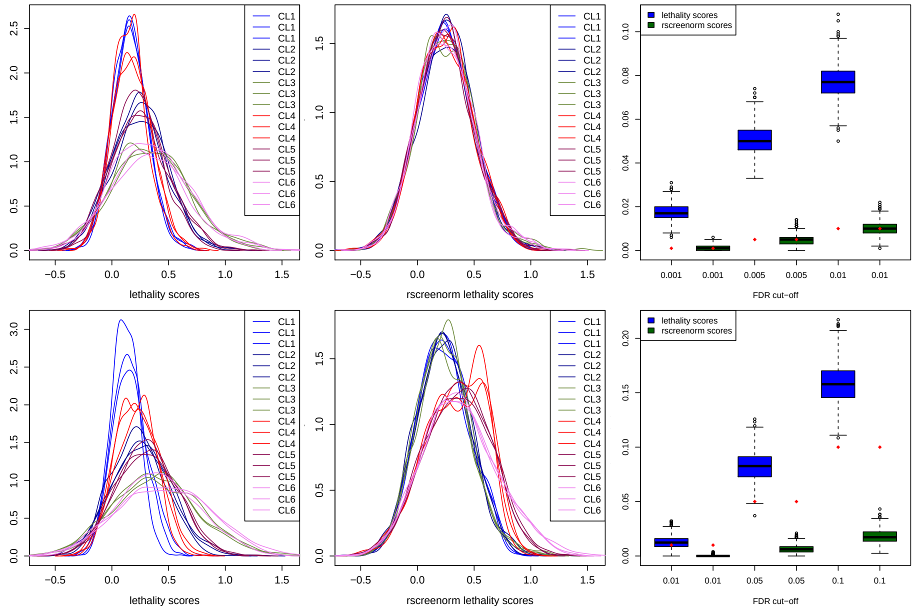

Figure S1: Simulation study: results without an effect between cell lines 1, 2, 3 and 4, 5, 6 (top-row graphs) and with an effect (bottom-row graphs). Graphs on first column represent density plots per replicate for each cell line, before normalization. Graphs on middle column represent density plots per replicate for each cell line, after rscreenorm, where we noticed that biological effects (cell lines in warmer colours vs. cell lines in colder colours) remain in the bottom graph, whilst no differences between cell lines are visible when no biological effect is present (top graph). Graphs on the right column display the proportion of false discoveries made across 1000 simulated datasets using the same setup, according to various false discovery-rate cut-offs, indicated by the red diamonds. Here we used different cut-offs for the no-effects and with-effects simulations for clarity, although conclusions are unchanged if cut-offs change.

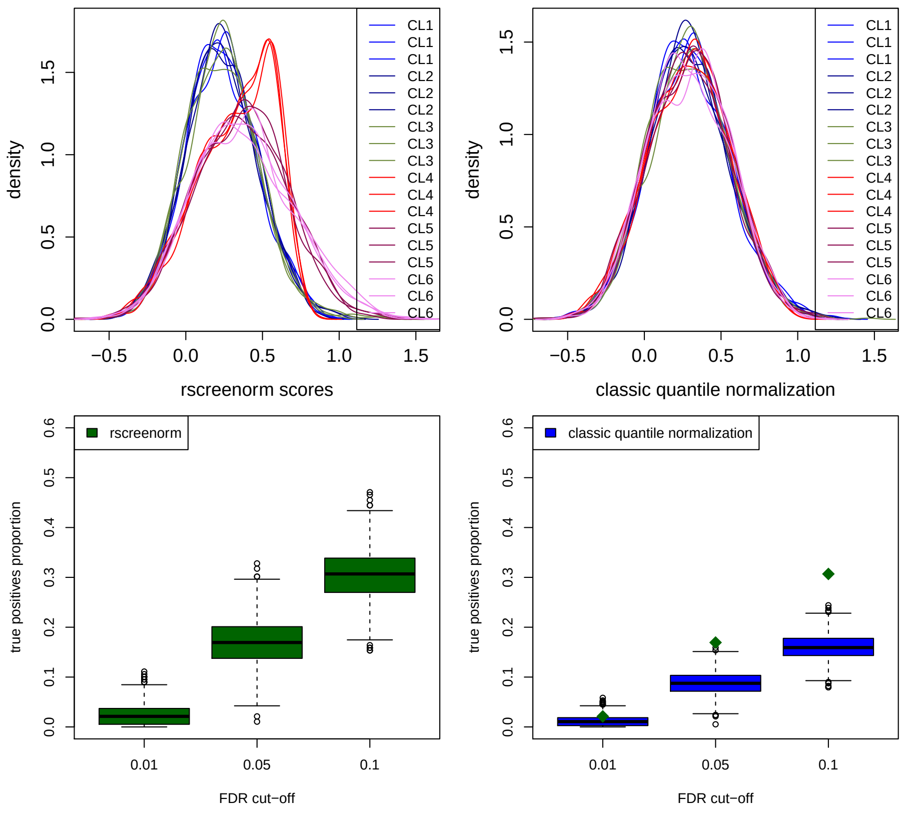

Figure S2: Simulation study results with effect between cell lines 1, 2, 3 and 4, 5, 6. Graphs on first row represent density plots per replicate for each cell line: on the left after rscreenorm, where we noticed that biological effects (cell lines in warmer colours vs. cell lines in colder colours) remain and, on the right, after classic quantile normalization using all library scores, where differences in the upper-tails corresponding to lethal hits have been corrected away. Graphs on the bottom row display the proportion of true discoveries made across 1000 simulated datasets using the same setup, according to various false discovery-rate cut-offs (left, rscreenorm data; right, classic quantile-normalized data). Median true positive rates obtained with rscreenorm are displayed in the right-hand side graph as green diamonds.

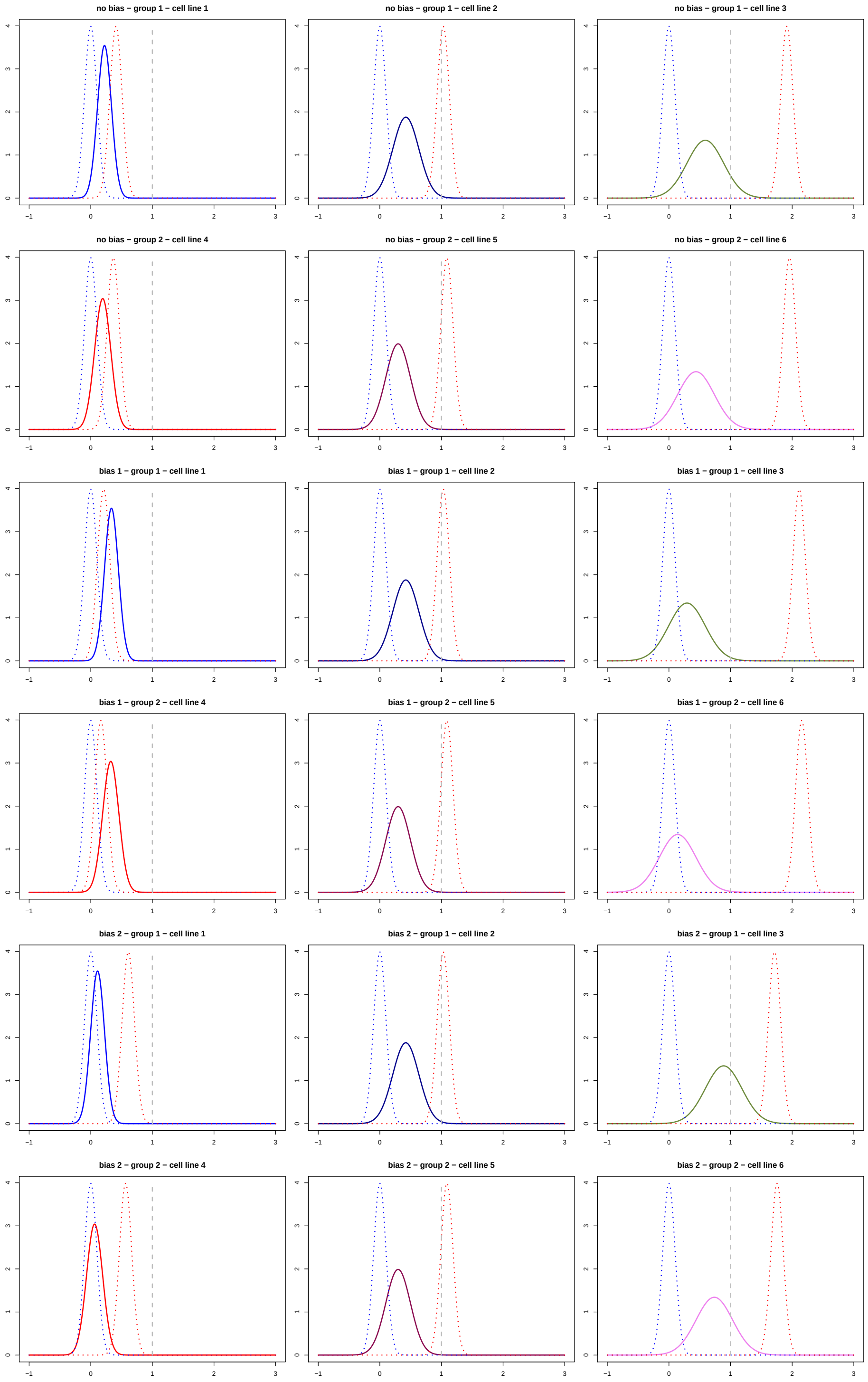

Figure S3: Simulation study: density of library feature means (solid line), and distributions of negative and positive controls (blue and red dotted lines, respectively), with one cell line per graph. Vertical gray-dashed line represents the response expected for positive controls. Rows 1, 2: stretch effect, but no positive control bias. Rows 3, 4: positive control bias 1. Rows 5, 6: positive control bias 2. The main difference between the no-bias and bias 1 situations are for cell lines 1, 4, which display larger lethality scores in bias 1 than without any bias.

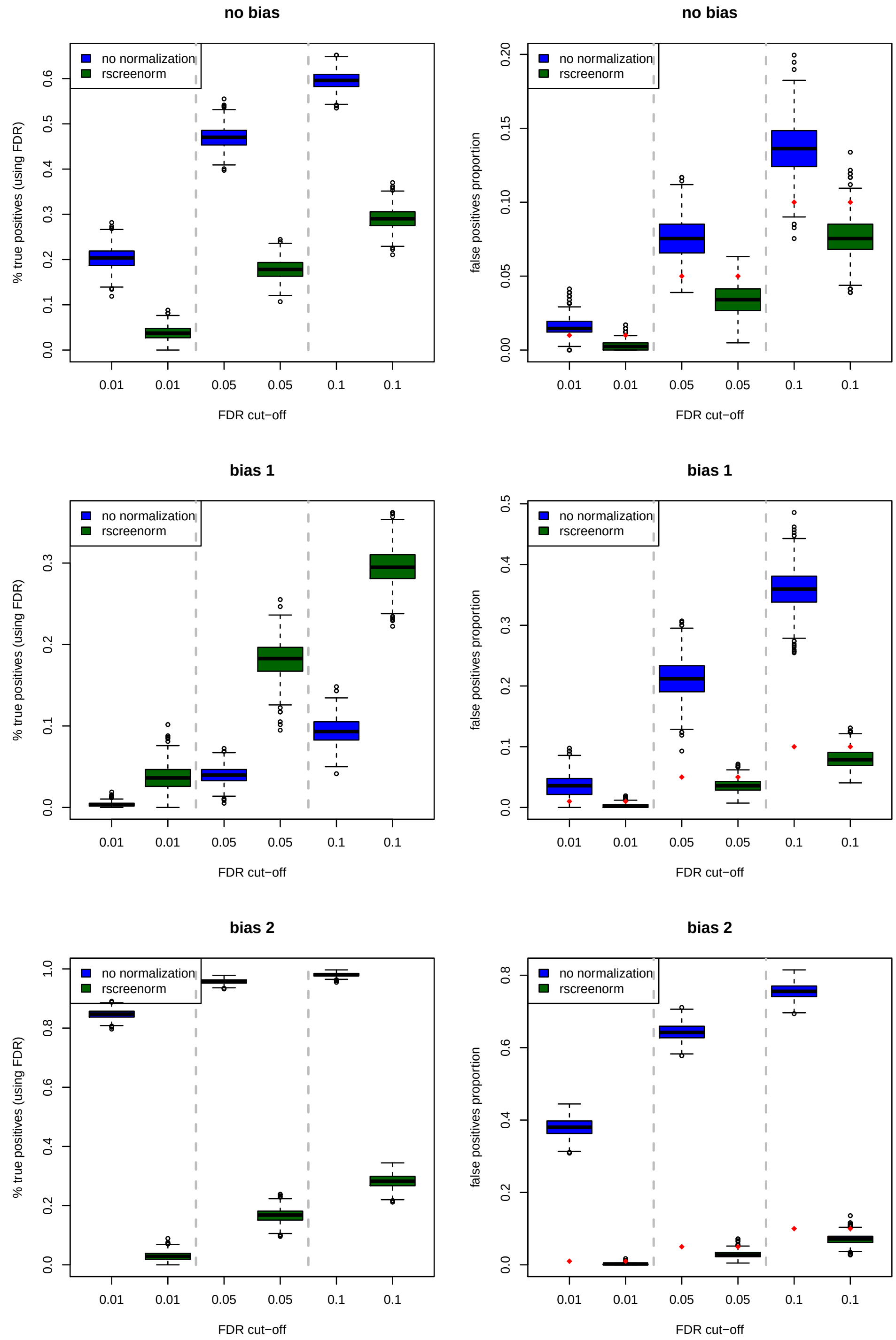

Figure S4: Simulation study: boxplots of true positive percentages (left column) and false positive proportions (right column) under the three bias-related situation: no bias (top row), bias 1 (middle row) and bias 2 (bottom row). Vertical gray-dashed lines separate results obtained for different FDR cut-offs in each graph.

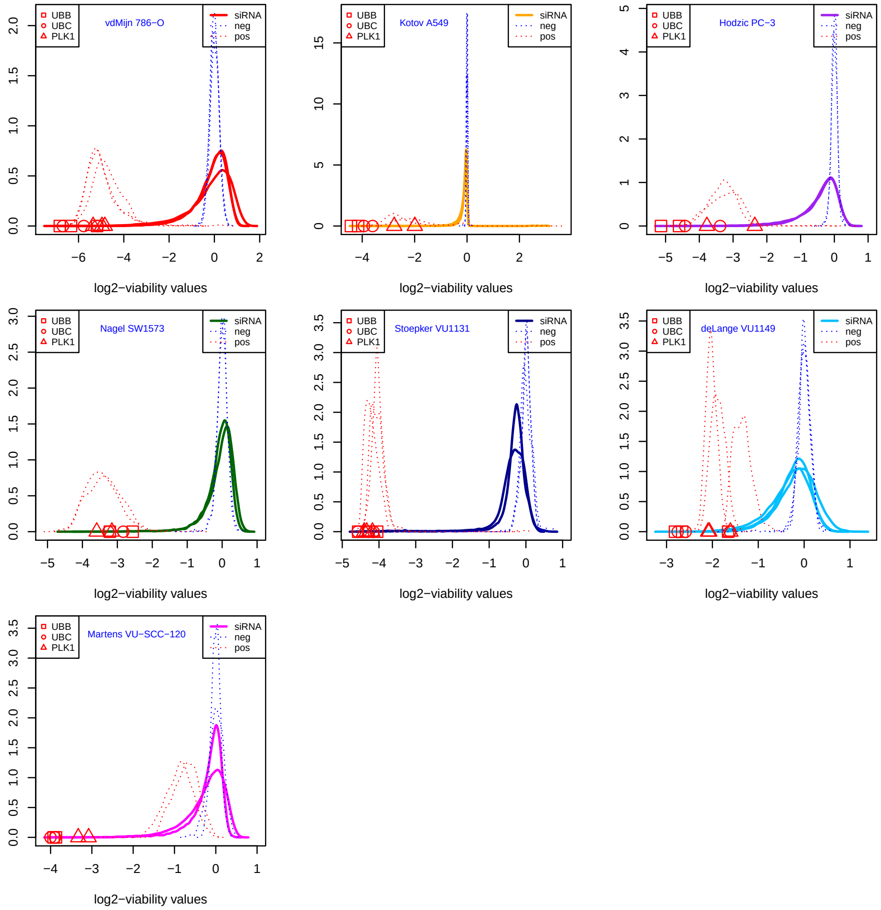

Figure S5: siRNA screen data example: density plots of siRNA data centered around siCons per plate, per experiment. Dotted lines represent distributions for controls: negative (blue) and positive (red). Values for library siRNAs siUBB, siUBC and siPLK1 are displayed as red squares, circles and triangles, respectively. These consistently display lethal phenotype across all cell lines and replicates, but yield different values depending on the cell line: for example, for cell lines 786-O and VU1131 these all yield log2-viabilities below -4 for all replicates, whilst for SW1573 and VU-SCC-120 their log2-viabilities are between -4 and -2.

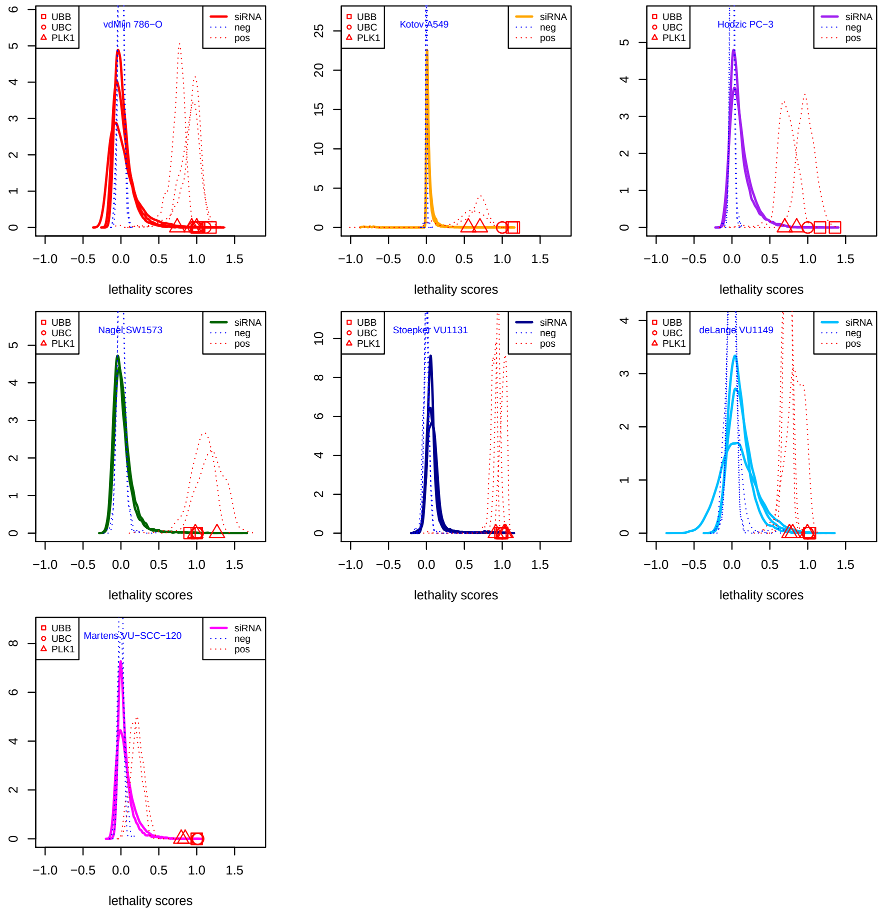

Figure S6: siRNA screen data example: density plots of lethality scores for all siRNAs, per experiment. Dotted lines represent distributions for controls: negative (blue) and positive (red). Values for library siRNAs siUBB, siUBC and siPLK1 are displayed as red squares, circles and triangles, respectively. These consistently display lethal phenotype across all cell lines and replicates, but yielded log2-viabilities that varied considerably between cell lines (see figure 5). In contrast, their lethality scores are much more comparable, being all between 0.5 and 1.5.

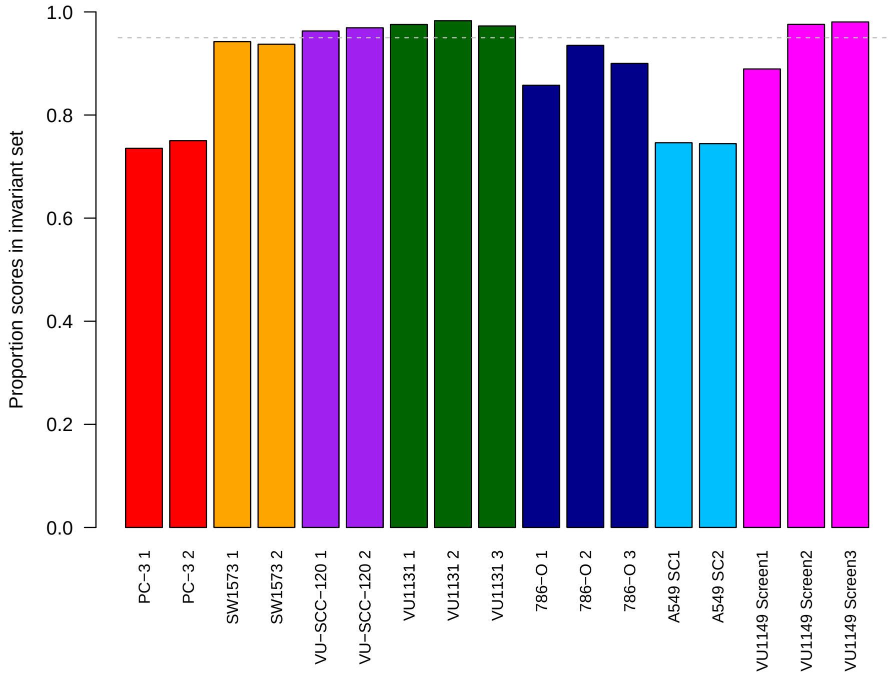

Figure S7: siRNA screen data example: proportions of lethality scores included in the core set for normalization, per replicate, when using $\gamma = 1$. The dashed line represents 95%.

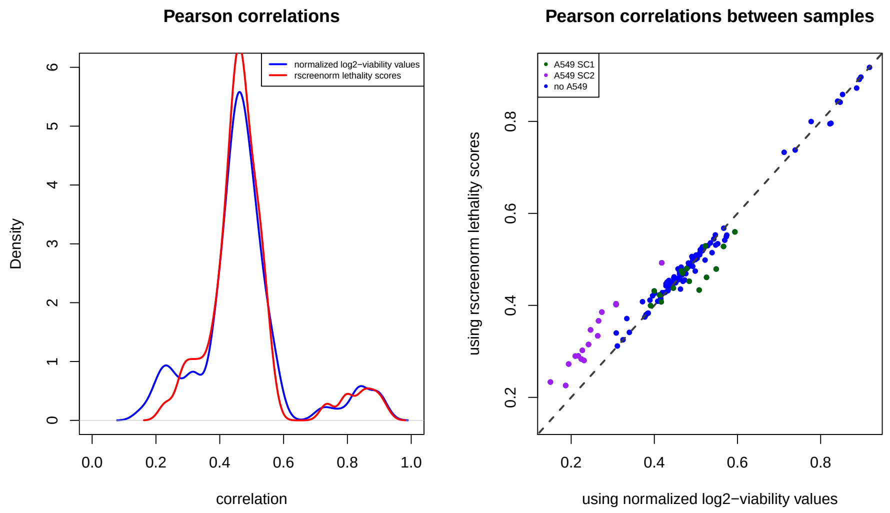

Figure S8: siRNA screen data example: Pearson correlation between pairs of replicates, both of the same cell line as from different cell lines. Left: Density of Pearson correlations, before (blue) and after (red) rscreenorm. Right: Pearson correlations between replicates before (x-axis) and after (y-axis) rscreenorm, sorted by their values before normalization. Correlations involving one of the cell line A549 replicates are coloured as either purple (replicate 1) or green (replicate 2). All correlations that changed by more than 0.05 between the two lists involve one of the A549 replicates.

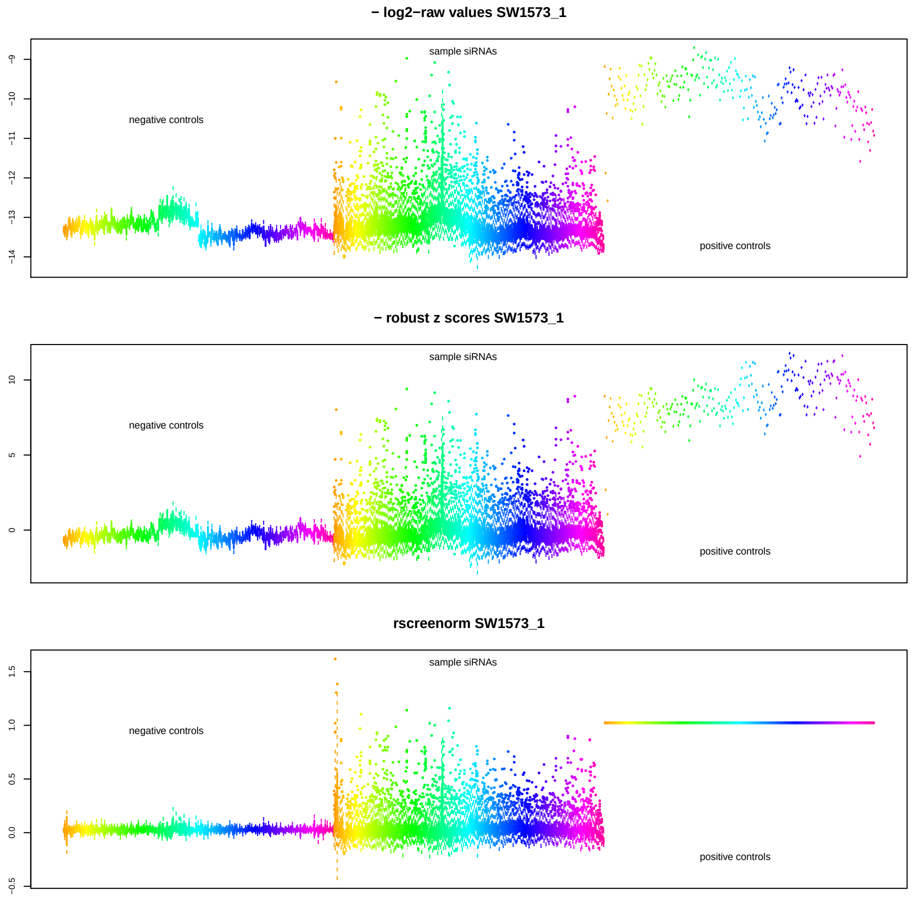

Figure S9: siRNA screen data example: Removal of plate effects within replicates of arrayed screens by rscreenorm. Boxplots of log2-raw viability values (top), robust z-scores (middle) and rscreenorm scores (bottom) for the first replicate of cell line SW1573. Same plates are identified by the same colours for negative controls (left boxes), sample siRNAs (middle boxes) and positive controls (right boxes). Here we display -log2-raw data and -robust z-scores, to make displays interpretable in the same way and direction so in all cases, values indicating more lethality are displayed higher than those corresponding to more viability.

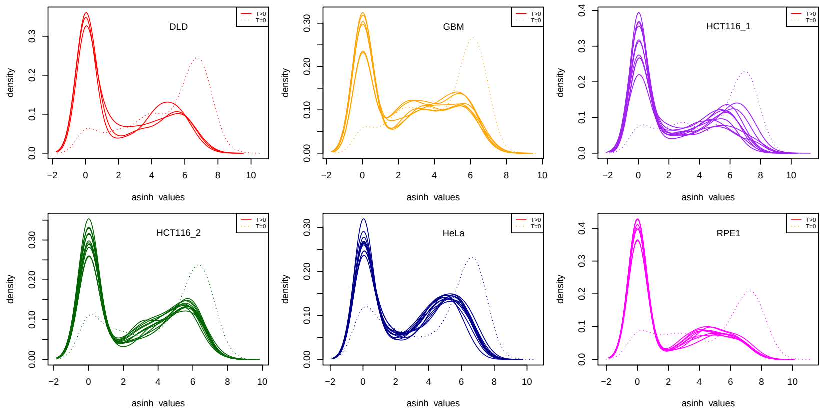

Figure S10: CRISPR-Cas screen data example: density plots of hyperbolic-arc sine-transformed viabilities for positive controls per replicate, grouped by cell line. Note that the distributions are always bimodal, with the one corresponding to $T = 0$ displaying a mode on the right-hand side, whilst the ones corresponding to $T > 0$ displaying modes on the left-hand side.

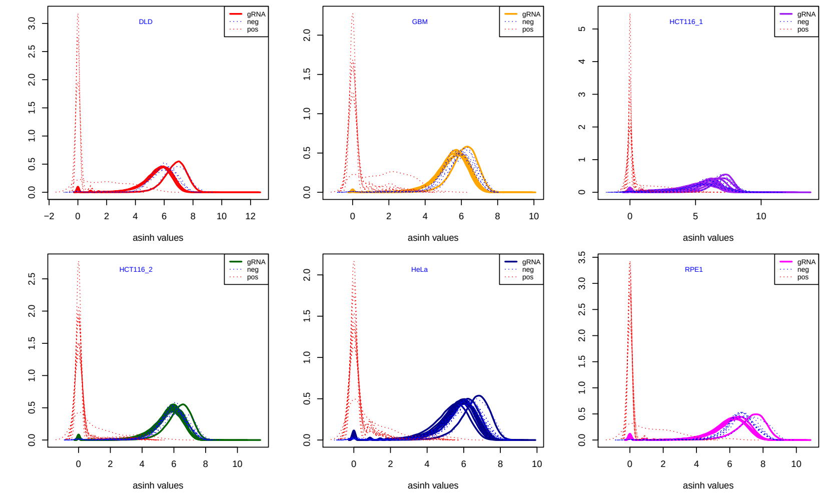

Figure S11: CRISPR-Cas screen data example: density plots of hyperbolic-arc sine-transformed viabilities per replicate, separately for library guide RNAs, negative and positive controls, with replicates grouped by cell line. Note that positive controls distributions are still bimodal for $T = 0$, but all others consistently represent lethal phenotypes.

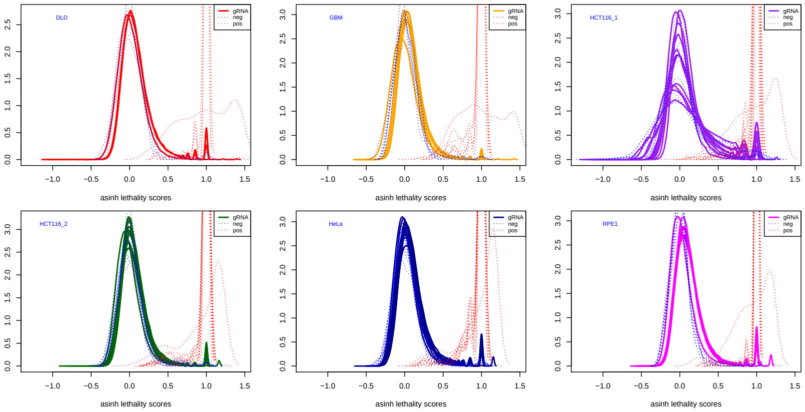

Figure S12: CRISPR-Cas screen data example: density plots of lethality scores per replicate, separately for library guide RNAs, negative and positive controls, with replicates grouped by cell line.

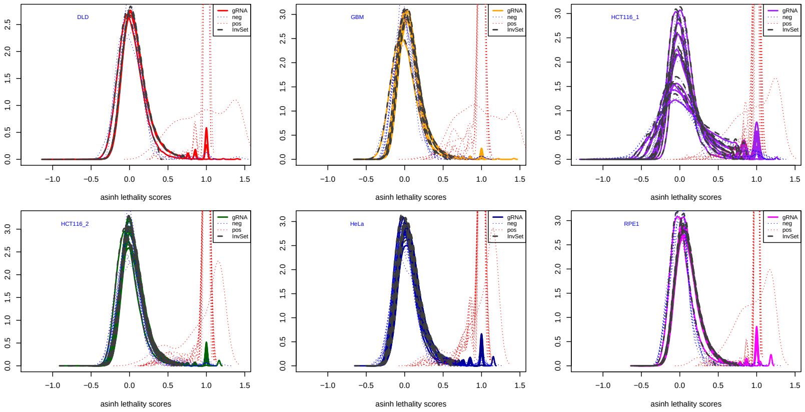

Figure S13: CRISPR-Cas screen data example: density plots of lethality scores per replicate, separately for library guide RNAs, negative and positive controls, with replicates grouped by cell line. Dark-gray dashed line: core set of 95% of all lethality scores used by rscreenorm.

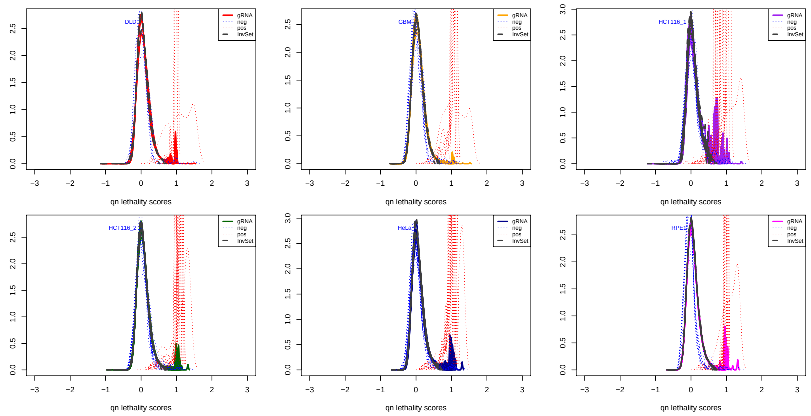

Figure S14: CRISPR-Cas screen data example: density plots of rscreenorm scores per replicate, separately for library guide RNAs, negative and positive controls, with replicates grouped by cell line.

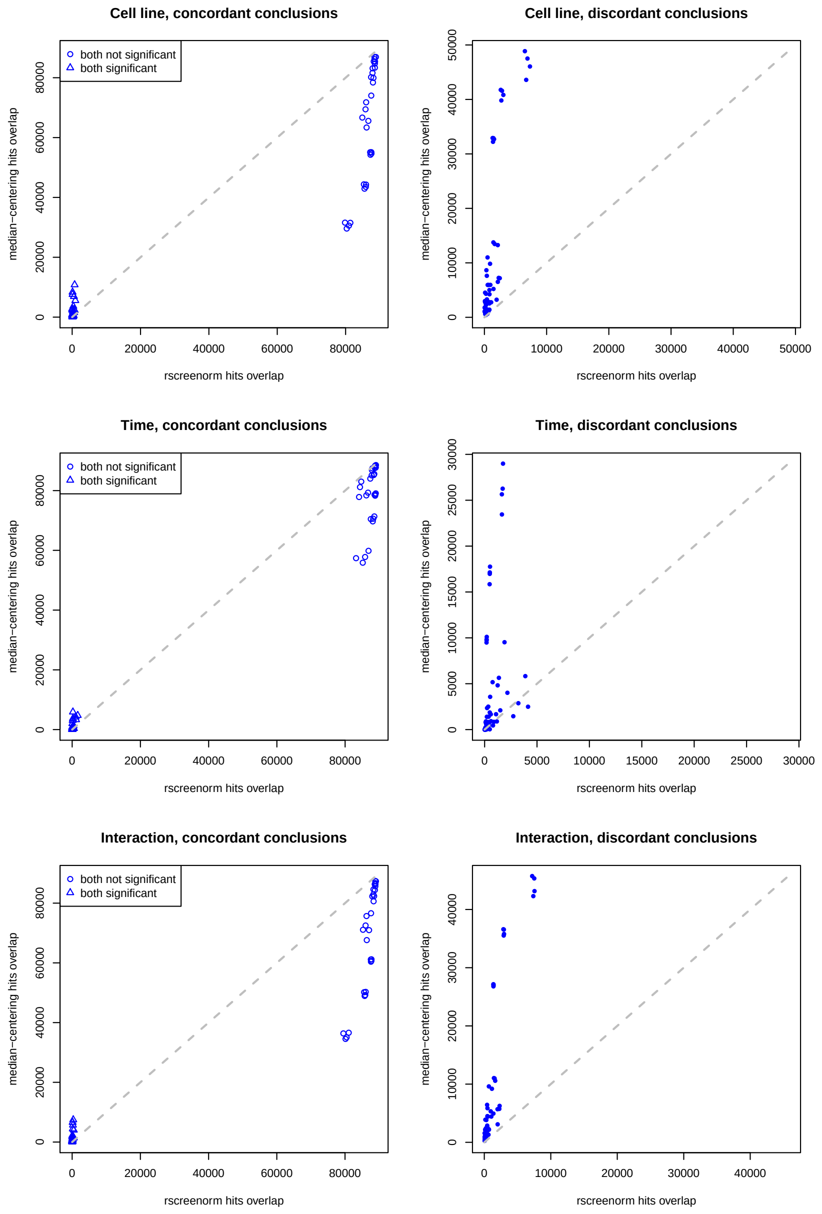

Figure S15: CRISPR-Cas screen data example: scatterplots of concordance (left) and discordance (right) counts per pre-processing, with rscreenorm on the x-axis and median-centering on the y-axis. Top graphs: cell line effect results. Middle graphs: time effect results. Bottom graphs: interaction effect between time and cell line.
